# Supplementary figures and images for: lncreased risk of slippage upon disengagement of the mitotic checkpoint
Source: PLoS Comput Biol. 2025 Mar 19;21(3):e1012879. doi: 10.1371/journal.pcbi.1012879 (PMC11981154; doi:10.1371/journal.pcbi.1012879)

Figure S1

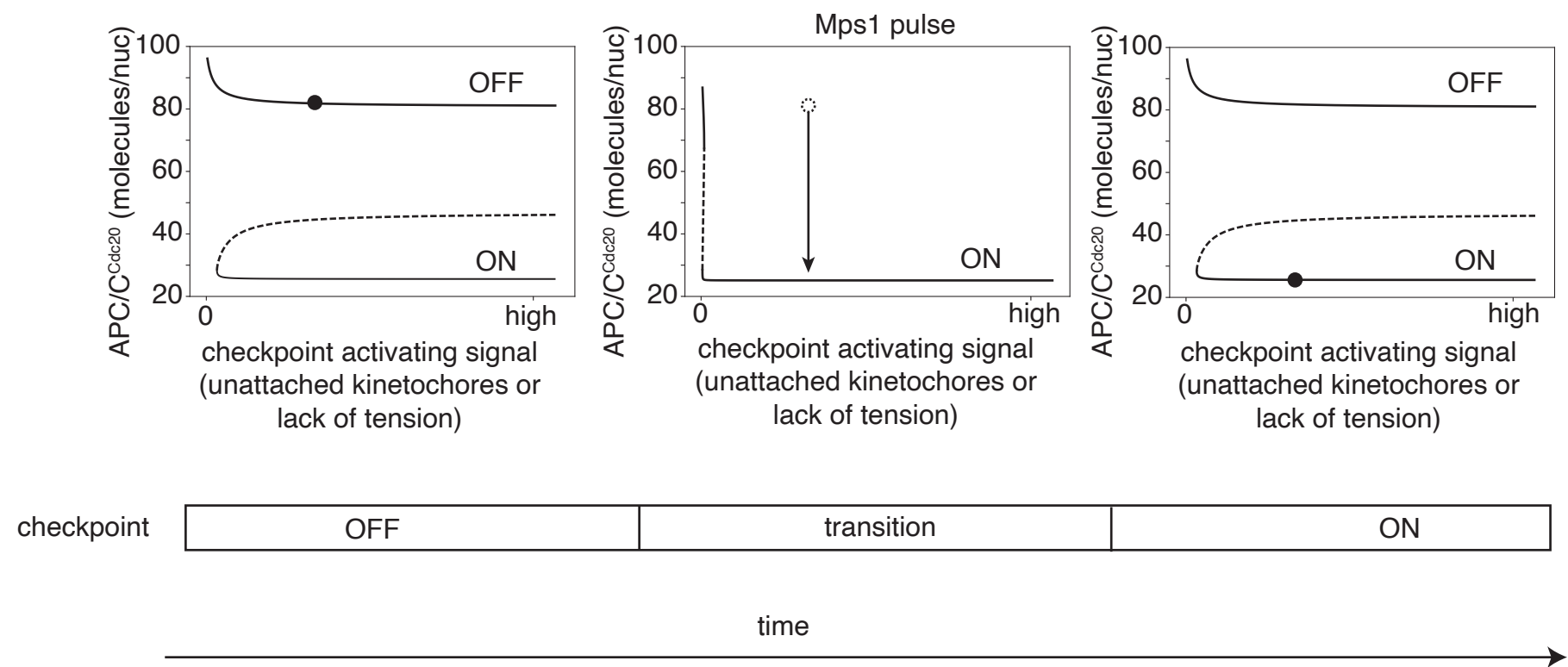

Supplement: S1 Fig — From left to right, bifurcation diagram for three consecutive moments of the experiment simulated in Fig 1D. At the beginning of the experiment (left), cells are in the checkpoint OFF state, arrested in anaphase (by the cdc15-2 mutation). Although all kinetochores are attached, on the x-axis the checkpoint activating signal is not zero due to the lack of tension between sister chromatids. When Mps1 is overexpressed (middle), the checkpoint OFF state is greatly reduced, and the system is attracted to the only available steady state, checkpoint ON. After the overexpression stops (right), the original checkpoint OFF steady state is available again. Yet, cells remain attracted to the checkpoint ON steady state. (PDF) [file pcbi.1012879.s001.pdf]

Figure S2

A

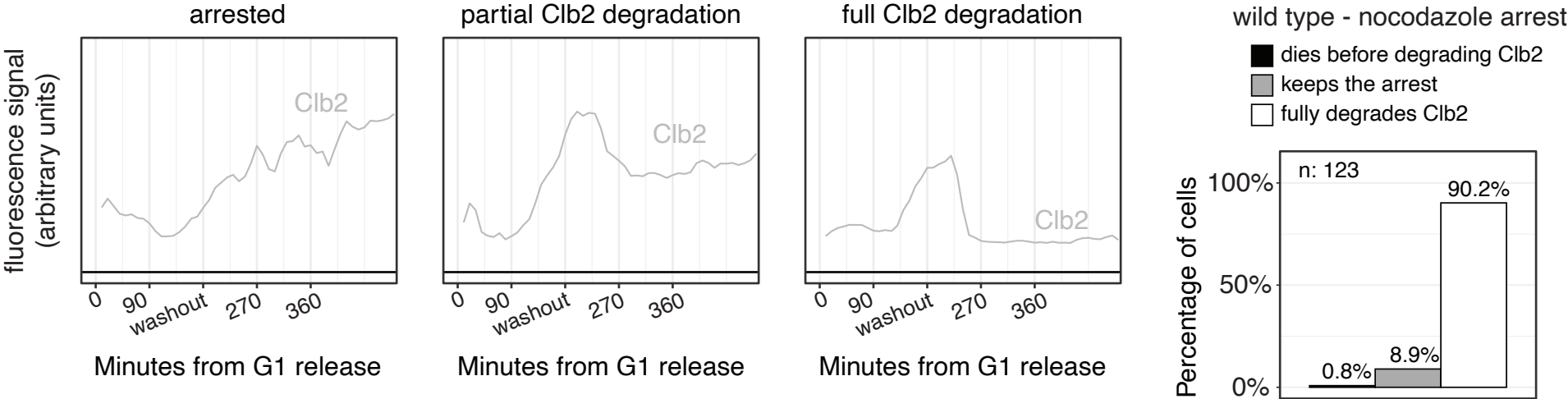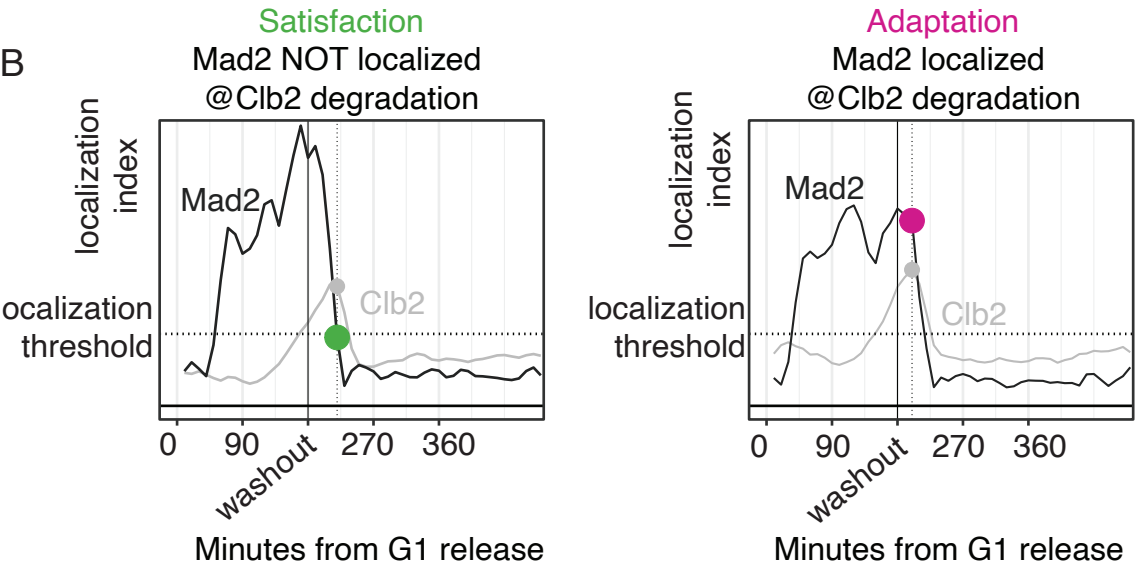

Supplement: S2 Fig — A) Classification of cell behaviors based on Clb2 dynamics. From left to right, a cell defined as arrested, where Clb2 signal is never degraded; a cell partially degrading Clb2; and a cell fully degrading Clb2. Rightmost panel: barplots presenting the percentages of the different classes of cells in wild-type cells arrested in nocodazole indefinitely. B) Two examples of Mad2 Localization Index (black curve) and Clb2 mean signal (grey curve) over time. In the left panel, a cell defined as exiting: at the moment of Clb2 degradation (grey dot, dashed vertical line) Mad2 Localization Index lies below the threshold (dashed horizontal line). The threshold is defined based on the Localization Index value in G1. The green dot highlights that the value of Mad2 Localization Index at Clb2 degradation time is below the threshold. In the right panel, a cell defined as adapting. In this cell, Mad2 Localization value at the Clb2 degradation time is above the threshold. This is highlighted by the purple dot. These examples come from a washout experiment but the definition applies also to cells always kept in nocodazole. Details of the analysis are presented in the Image analysis section of the Materials and Methods. (PDF) [file pcbi.1012879.s002.pdf]

Figure S3

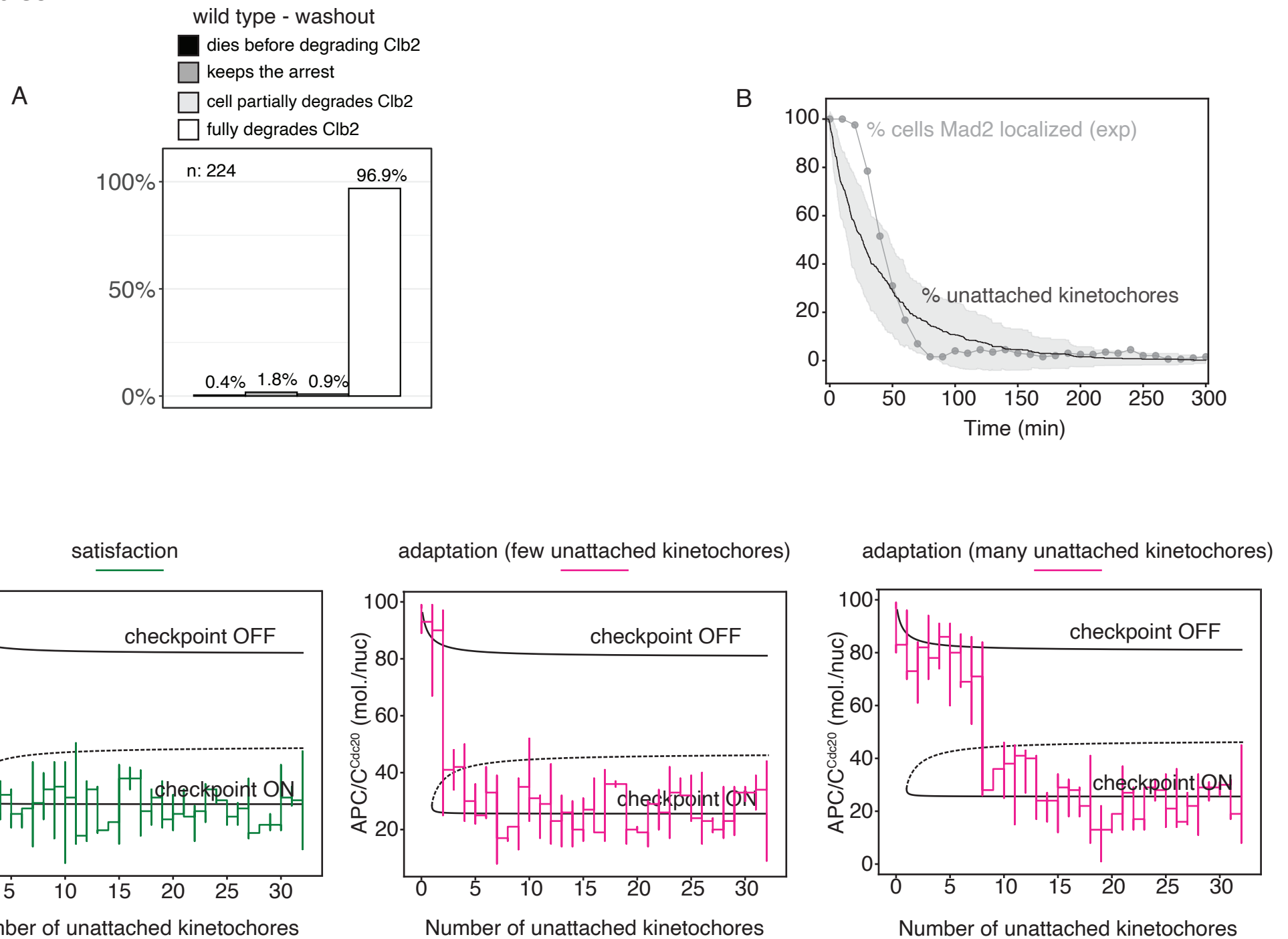

Supplement: S3 Fig — A) Barplot presenting the percentages of the different classes of Clb2 behavior in wild-type cells arrested in nocodazole for 180 minutes. For the definition of the cell classes see S2A Fig. B) Comparison between experimental and simulated values for kinetochores-attachment dynamics. Experimental data show the dynamics of cells with Mad2 Localization Index above the threshold over. Simulated data show the dynamics of unattached kinetochores (percentage of the number of unattached kinetochores per cell). C) Bifurcation diagrams with APC/CCdc20 at steady state in function of the number of unattached kinetochores superimposed with the trajectories of attachment (Fig 3B). Representative trajectories are shown for exit (left), and adaptation with few (center) or many (right) unattached kinetochores. (PDF) [file pcbi.1012879.s003.pdf]

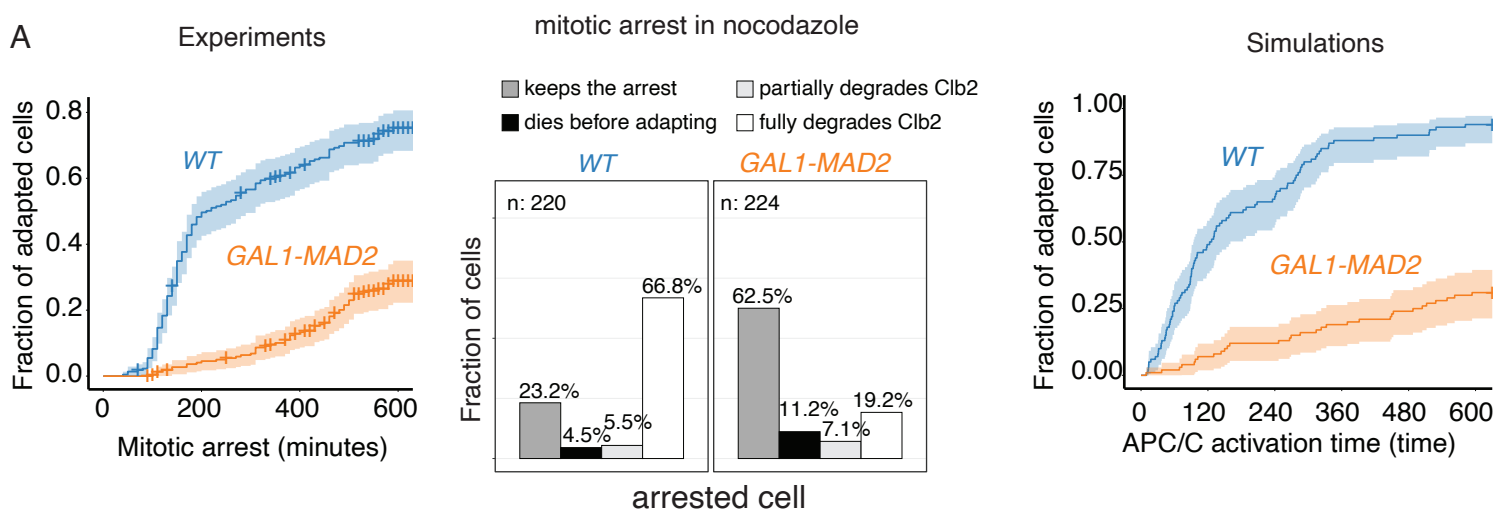

Figure S5

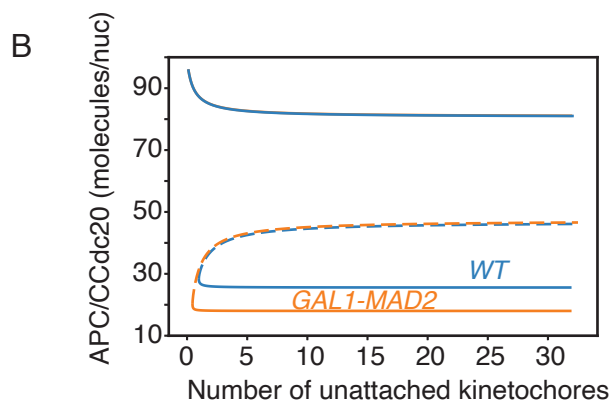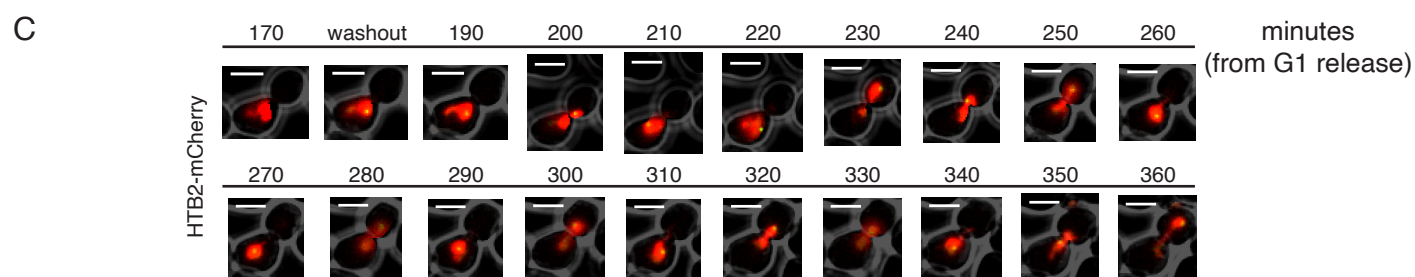

**D** proper chromosome segregation

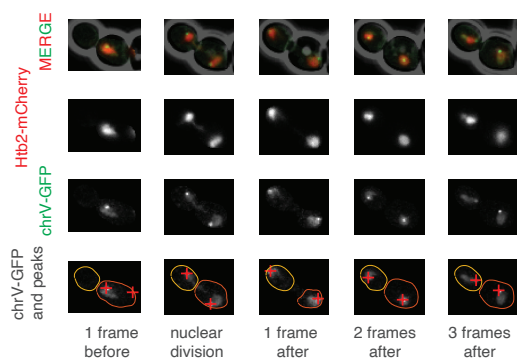

**E** chromosome missegregation

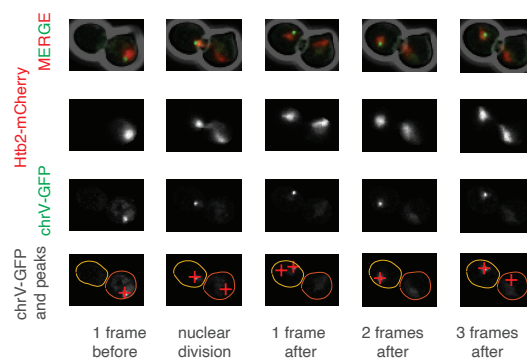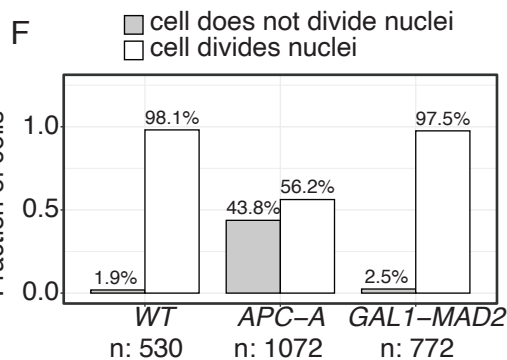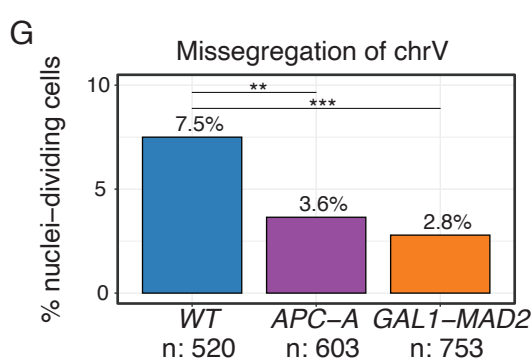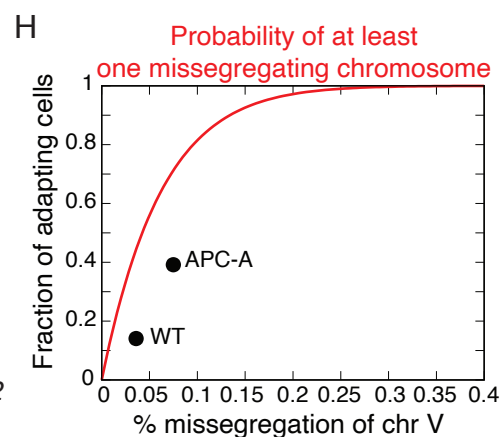

Supplement: S5 Fig — A) Left panel: Cumulative distribution of length of mitotic arrest in wild-type or Mad2-overepressing cells. Cells growing in YPRG are arrested in G1 and released in nocodazole, indefinitely. Cells carry Mad2-GFP to record checkpoint activation status, and Clb2-mCherry to record both mitotic entry (Clb2 rise time) and mitotic exit (Clb2 degradation time). Mitotic arrest is defined as the difference between mitotic exit and mitotic entry. Central panel: barplots with the percentages of the different classes of Clb2 behavior in wild-type or GAL1-Mad2 cells arrested indefinitely in nocodazole. For the definition of the cell classes see S2A Fig. Right panel: cumulative distribution of APC/C activation time for the simulated data. Adaptation is defined as APC/C crossing the activation threshold with at least one unattached kinetochore. Reactions in Table 3, parameters in Table 2, initial conditions in Table 1 (checkpoint ON). B) Bifurcation diagrams of wild type cells (same as in Fig 1C) and GAL1-MAD2 mutant. Expression level of Mad2 is increased to 125% compare to WT. In GAL1-MAD2, the saddle node bifurcation is located at 0.4 unattached kinetochores. Equations in S1 Text, and parameters in Table 2. C) Example of an arrested cell taken from a movie of cells growing in YPRG, arrested in G1 and released in nocodazole for 180 minutes, when nocodazole is removed from the media. Cells carry the tetR-GFP/tetO construct to keep track of chrV segregation, and Htb2-mCherry to score nuclear division time. Definition of nuclear division time is presented in the Image analysis section of the Materials and Methods. D) Example of a cell correctly segregating chrV. In the lowest row, yellow and orange traces are cells segmentations, while red crosses mark the position of chrV. In the second column is the frame when nuclear division is registered. Definitions of correct or wrong segregation are presented in the Image analysis section of the Materials and Methods. E) Example of a cell m [file pcbi.1012879.s005.pdf]

Figure S6

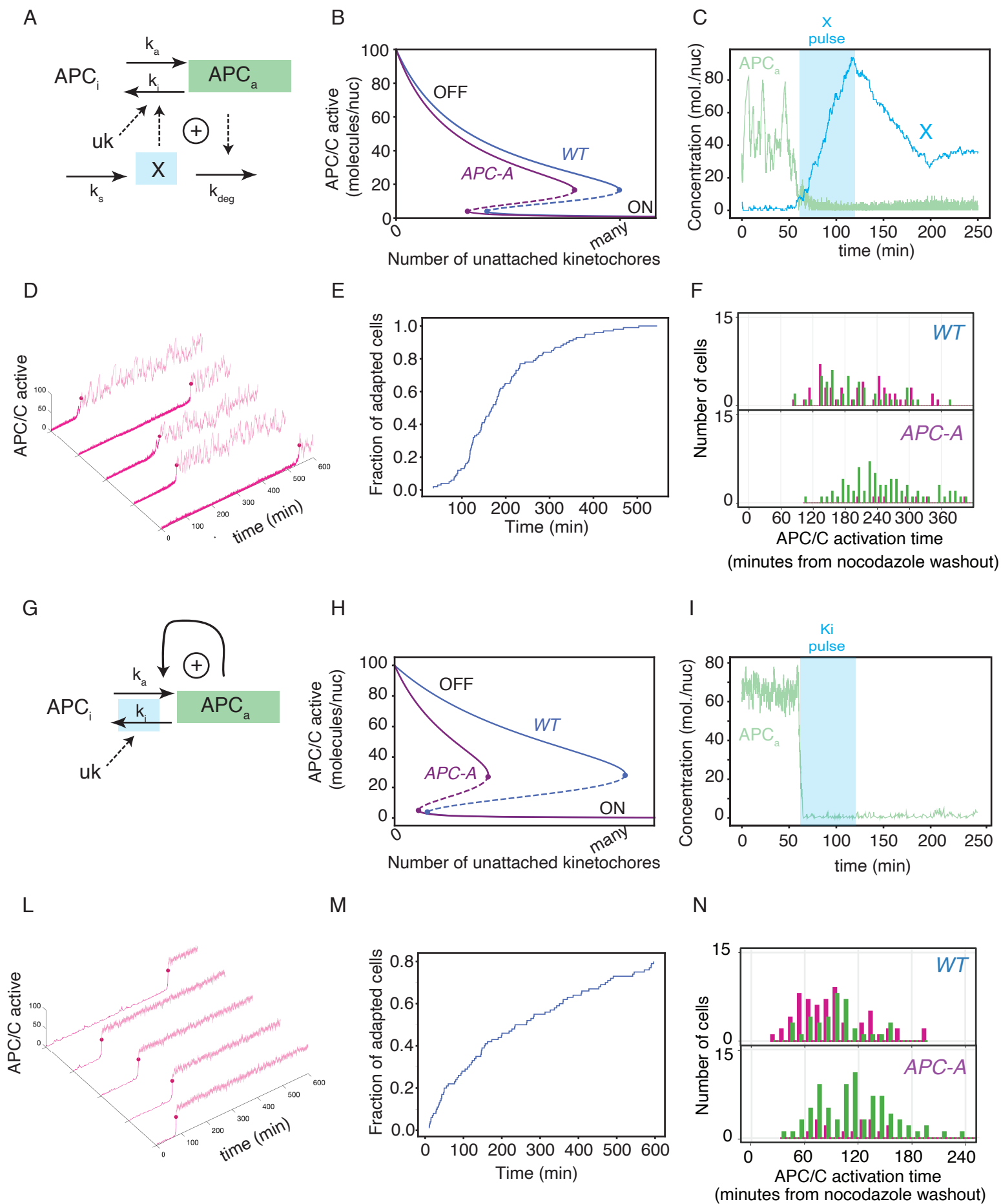

Supplement: S6 Fig — A) Wiring diagram of simple Model 1, which belongs to the class shown in Fig 1A left. B) The model shows bistability. It also reproduces the alteration in the bifurcation diagram of APC/C similarly to what seen for the detailed model in Fig 4A. C) Simulation of the Palframan’s experiment[9] to show bistability in the simple model, in analogy with Fig 1D. D) Stochastic simulations with fixed number of unattached kinetochores give rise to the exponential distribution shown in (E), in analogy with Fig 2D and 2E. F) APC-A mutants decrease the frequency of adaptations upon washout, similarly to Fig 4C. G) Wiring diagram of simple Model 2, which belongs to the class shown in Fig 1A right. H) The model shows bistability. It also reproduces the alteration in the bifurcation diagram of APC/C similarly to what seen for the detailed model in Fig 4A. I) Simulation of the Palframan’s experiment [9] to show bistability in the simple model, in analogy with Fig 1D. L) Stochastic simulations with fixed number of unattached kinetochores give rise to the exponential distribution shown in (M), in analogy with Fig 2D and 2E. N) APC-A mutants decrease the frequency of adaptations upon washout, similarly to Fig 4C. (PDF) [file pcbi.1012879.s006.pdf]
