# Supplementary material for: lncreased risk of slippage upon disengagement of the mitotic checkpoint
Source: PLoS Comput Biol. 2025 Mar 19;21(3):e1012879. doi: 10.1371/journal.pcbi.1012879 (PMC11981154; doi:10.1371/journal.pcbi.1012879)
Supplement: S4 Fig — A) Bifurcation analysis shows how increasing the distance between stable (checkpoint ON) and unstable steady states minimizes the effects of random fluctuations. B) Absolute concentrations of APC/CMCC and total MCC as measured by Fluorescence Cross-Correlation Spectroscopy (FCCS). Wild-type or APC-A cells are arrested in G1 and released in nocodazole. Cells are measured in nocodazole between 150 and 210 minutes. To measure APC/CMCC, we measured the cross-correlation of Mad2-GFP and Cdc23-mCherry, while for total MCC we used Mad2-GFP and Mad3-mCherry. To compare the distribution of concentrations we used a linear model adjusting for batch effects for experiments performed on different days. p-values are 0.81 for APC/CMCC and 2.7 10-6 for MCC total. Details of treatment, measurements, and analyses are presented in the FCCS section of Materials and Methods. C) Left panel: barplot presenting the percentages of the different classes of Clb2 behavior in APC-A cells arrested in nocodazole indefinitely. For the definition of the different classes see S2A Fig. Central panel: cumulative distribution of length of mitotic arrest in wild-type or APC-A cells. Wild-type curve is the same presented in Fig 2B. Cells growing in YPD are arrested in G1 and released in nocodazole, indefinitely. Cells carry Mad2-GFP to record checkpoint activation status, and Clb2-mCherry to record both mitotic entry (Clb2 rise time) and mitotic exit (Clb2 degradation time). The duration of mitotic arrest is defined as the difference between mitotic exit and mitotic entry. Right panel: cumulative distribution of APC/C activation time for the simulated data. Adaptation is defined as APC/C crossing the activation threshold with at least one unattached kinetochore. Reactions in Table 3, parameters in Table 2, initial conditions in Table 1 (checkpoint ON). D) Barplot presenting the percentages of the different classes of Clb2 behavior in APC-A cells arrested in nocodazole for 180 minutes. For the definition [file pcbi.1012879.s004.pdf]

Figure S4

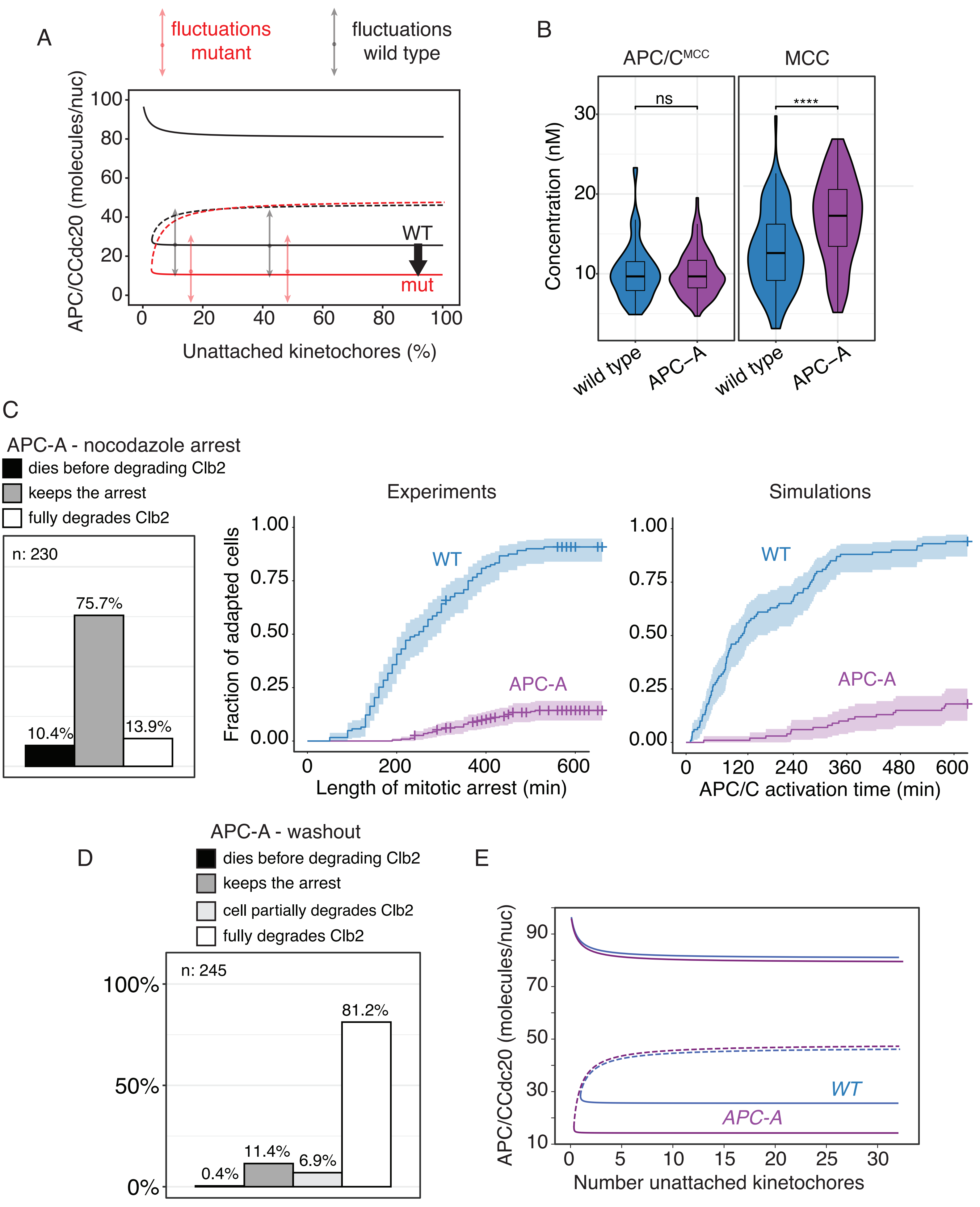
A
fluctuations mutant
fluctuations wild type
APC/CCdc20 (molecules/nuc)
100
80
60
40
20
0
WT
mut
0
20
40
60
80
100
Unattached kinetochores (%)
B
APC/CMCC
MCC
ns
****
Concentration (nM)
30
20
10
wild type
APC–A
wild type
APC–A
C
APC-A - nocodazole arrest
dies before degrading Clb2
keeps the arrest
fully degrades Clb2
n: 230
75.7%
10.4%
13.9%
Experiments
Simulations
Fraction of adapted cells
1.00
0.75
0.50
0.25
0.00
WT
APC-A
0
200
400
600
Length of mitotic arrest (min)
0
120
240
360
480
600
APC/C activation time (min)
D
APC-A - washout
dies before degrading Clb2
keeps the arrest
cell partially degrades Clb2
fully degrades Clb2
n: 245
100%
50%
0%
0.4%
11.4%
6.9%
81.2%
E
APC/CCdc20 (molecules/nuc)
90
70
50
30
10
WT
APC-A
0
5
10
15
20
25
30
Number unattached kinetochores
