# Supplementary material for: lncreased risk of slippage upon disengagement of the mitotic checkpoint
Source: PLoS Comput Biol. 2025 Mar 19;21(3):e1012879. doi: 10.1371/journal.pcbi.1012879 (PMC11981154; doi:10.1371/journal.pcbi.1012879)
Supplement: S1 Table — (PDF) [file pcbi.1012879.s009.pdf]

Table S1. List of strains used in this study

Strain number	genotype	figure
3812	MATa Mad2-3GFP::KanMX6 Clb2-3mCherry-dcu::hphNT1	Mad2Clb2 WT
3964	MATa Mad2-3GFP::KanMX6 Clb2-3mCherry-dcu::hphNT1 CDC16-6A::TRP1 CDC27-5A::KAN	Mad2Clb2 APC-A
4000	MATa Mad2-3GFP::KanMX6 Clb2-3mCherry-dcu::hphNT1 leu2-3::LEU2::GAL1-MAD2 (single copy)	Mad2Clb2 GAL-Mad2
4013	MATa his3-11,15::HIS3tetR-GFP ura3::3XURA3tetO112 HTB2-Cherry::HIS3	missegr
4152	MATa his3-11,15::HIS3tetR-GFP ura3::3XURA3tetO112 HTB2-Cherry::HIS3 leu2-3::LEU2::GAL1-MAD2 (single copy)	missegr
4156	MATa his3-11,15::HIS3tetR-GFP ura3::3XURA3tetO112 HTB2-Cherry::HIS3 CDC16-6A::TRP1 CDC27-5A::KAN	missegr
2932	MATa ADE2 Cdc23-3mCherry-dcu::hphNT1 Apc5-3myeGFP-dcu::NAT	FCCS APC total
2954	MATa ADE2 Cdc23-3mCherry-dcu::hphNT1 Cdc16-3myeGFP-dcu::NAT	FCCS APC total
2886	MATa ADE2 Mad2-3myeGFP-dcu::NAT Cdc23-3mCherry-dcu::hphNT1	FCCS ACP:MCC
3312	MATa ADE2 Mad2-3myeGFP-dcu::NAT Cdc23-3mCherry-dcu::hphNT1 CDC16-6A::TRP1 CDC27-5A::KAN	FCCS ACP:MCC
3268	MATa ADE2 Mad2-3myeGFP-dcu::NAT Mad3-3mCherry-dcu::hphNT1	FCCS MCC
3346	MATa ADE2 Mad2-3myeGFP-dcu::NAT Mad3-3mCherry-dcu::hphNT1 CDC16-6A::TRP1 CDC27-5A::KAN	FCCS MCC
