# Supplementary material for: lncreased risk of slippage upon disengagement of the mitotic checkpoint
Source: PLoS Comput Biol. 2025 Mar 19;21(3):e1012879. doi: 10.1371/journal.pcbi.1012879 (PMC11981154; doi:10.1371/journal.pcbi.1012879)
Supplement: S1 Text — (DOCX) [file pcbi.1012879.s010.pdf]

Stier et al. Model's description

Deterministic model- equations

The deterministic version of the model can be translated into the following set of ordinary differential equations, involving 9 variables ($A, Mad_i, Mad_a, C, AC, MC, ACMC, Mps1, nUK$). Notice, in the text AC is reported as APC/CCdc20, A as APC/C, C as Cdc20, MC as MCC and ACMC as APC/CMCC: the name have been shortened for the sake of simplicity.

The model is defined by the following differential equations:

1. $Mps1$: this molecule is synthesized at a constant rate k_{syn_X} and degraded dependently (k_{degX}) and independently (k_{degBGX}) from APC/C^{Cdc20}:

$$\frac{dMps1}{dt} = k_{syn_X} - k_{deg_X} \cdot Mps1 \cdot AC - k_{deg_{BGX}} \cdot Mps1$$

2. Mad_i/Mad_a: with these variables, we include the checkpoint components that form the signaling pathway (Mad2, Mad3, Bub3). Activation of those components (the active form being Mad_a) requires the presence of Mps1 and unattached kinetochores. Both activation and inactivation are described as Michaelis-Menten kinetics (with the same constant *J*). Aravamudhan et al. have shown that the total number of recruited SAC proteins reaches saturation when ~10 signaling kinetochores are present in a cell [1]. Thus the fraction of active SAC components can be described with the formula $\frac{k' \cdot nUK}{J_n + nUK}$ where k' and Jn can be obtained from Figure 1E in [1] as described later. Mad_a can reversibly bind to Cdc20 (C in the model) to form the mitotic checkpoint complex (MC):

$$\frac{dMad_a}{dt} = k_{act} \cdot Mps1 \cdot \frac{k' \cdot nUK}{J_n + nUK} \cdot \frac{Mad_i}{J + Mad_i} - k_{inact} \cdot \frac{Mad_a}{J + Mad_a} - k_{assMC} \cdot Mad_a \cdot C + k_{dissMC} \cdot MC$$

3. APC/C^{Cdc20} (AC in the model) is formed by reversible binding of APC/C (A) and its coactivator Cdc20 (C). It also takes part in the formation of the inhibited APC complex ($ACMC$), associating with the mitotic checkpoint complex (MC). APC/C^{Cdc20} is degraded via background degradation (k_{degBG}). The differential equation for AC is:

$$\frac{dAC}{dt} = k_{assAC} \cdot A \cdot C - k_{dissAC} \cdot AC - k_{assACMC} \cdot AC \cdot MC + k_{dissACMC} \cdot ACMC - k_{degBG} \cdot AC$$

4. The Mitotic Checkpoint complex (MC in the model): this complex is created by reversible binding of Mad_a and C. Furthermore, MC forms from dissociation of the inhibited APC/C ($ACMC$) and is degraded similarly to AC via background degradation:

$$\frac{dMC}{dt} = k_{assMC} \cdot Mad_a \cdot C - k_{dissMC} \cdot MC - k_{assACMC} \cdot AC \cdot MC + k_{dissACMC} \cdot ACMC - k_{degBG} \cdot MC$$

5. The inhibited form of APC/C^{Cdc20} ($ACMC$ in the model) is formed by reversible binding of AC and MC. It is degraded both actively (k_{deg}) and via the background degradation (k_{degBG}).

$$\frac{dACMC}{dt} = k_{assACMC} \cdot AC \cdot MC - k_{dissACMC} \cdot ACMC - k_{deg} \cdot ACMC - k_{degBG} \cdot ACMC$$

6. Free Cdc20 (C in the model) is synthesized at a constant rate $k_{syn_{C20}}$ and participates in the formation of MC and AC (reversible reactions) and degraded via background degradation. The active degradation

results in one molecule of C per degraded $ACMC$ (thus only one of the two molecules of C involved in the formation of $ACMC$ is degraded).

$$\frac{dC}{dt} = k_{syn_{C20}} - k_{assAC} \cdot A \cdot C + k_{kdissAC} \cdot AC - k_{assMC} \cdot Mad_a \cdot C + k_{dissMC} \cdot MC + k_{deg} \cdot ACMC$$

$$-k_{degBG} \cdot C$$

7. Inhibited Mad (Mad_i): inactive Mad is produced by degradation of Cdc20 in MCC (either MCC or APC/MCC).

$$\frac{dMad_i}{dt} = -k_{act} \cdot Mps1 \cdot \frac{k' \cdot nUK}{J_n + nUK} \cdot \frac{Mad_i}{J + Mad_i} + k_{inact} \cdot \frac{Mad_a}{J + Mad_a} + k_{deg} \cdot ACMC + k_{kdegBG} \cdot \mathrm{ACMC}$$

$$+k_{degBG} \cdot MC$$

8. Free APC/C (A in the model):

$$\frac{dA}{dt} = -k_{assAC} \cdot A \cdot C + k_{dissAC} \cdot AC + k_{degBG} \cdot AC + k_{deg} \cdot ACMC + k_{degBG} \cdot ACMC$$

Since APC/C and Mad species are stable, we can derive free APC/C (A) and inactive Mad (Mad_i) from the following algebraic equations:

$$A = A_{total} - (AC + ACMC)$$

$$Mad_i = Mad_{total} - (Mad_a + MC + ACMC)$$

Kinetochore attachment dynamics

To reproduce data about drug-washout, we introduced a variable nUK (number of unattached kinetochores). We assume that the rate of attachment is proportional to the number of remaining unattached kinetochores ($k_{nUK_{att}} \cdot nUK$).

The rate constant $k_{nUK_{att}}$ was obtained by fitting the resulting kinetics of attachment to experimental data. A value of $0.03 min^{-1}$ gave simulations in line with the removal of Mad2 signal from kinetochores, as shown in Figure S3B.

Kinetochore signaling

Yeast cells treated with $15\ \mu g/\ ml$ nocodazole show between 8-10±2 unattached kinetochores. At around this value checkpoint signaling at kinetochores saturates [1]. $15\ \mu g/\ ml$ is the same drug concentration that was used in our experiments. For this reason in the model we saturate the checkpoint signal at around 10 unattached kinetochores using a hyperbolic relationship for the checkpoint signal= $\frac{k' \cdot nUK}{J_n + nUK}$.
In experiments, the Michaelis-Menten constant J_n, is ~3. The J_n has a direct effect on the position of the left saddle node of the bifurcation diagram, which in our model is crucial for the inactivation of the checkpoint. For the model we used J_n = 0.4, a value which allows the saddle node to be at $nUK\ = \sim 0.9$ (i.e., the checkpoint ON state exists until one unattached kinetochore).

APC/CCdc20 activation and mitotic arrest

Most experimental data track the time of Clb2 degradation by APC/CCdc20. In the model, this event is marked by the increase of APC/CCdc20 levels above an activation threshold, which we set to 80 molecules/cell see Figure 3B. Hence, the time elapsed between the beginning of the simulation and the time APC/CCdc20 crosses this threshold level corresponds to the time of mitotic arrest.

PARAMETER VALUES

Whenever possible we used experimental measurements to fix model parameters. For others we could at least give reasonable ranges, and their exact values were determined from fitting other experimental data with Sloppycell [2, 3] (see Methods for the deterministic model). Hereafter, we describe all parameters and experiments used to fit them.

Fixed Parameters

The parameters that we could determine directly based on experimental measurements were the total amounts for APC/C and Mad concentrations. Concentrations were measured in [4], either with western blotting or fluorescence correlation and cross-correlation spectroscopy (FCS and FCCS).

Total APC/C (A_{total}): The absolute concentration of APC/C was measured by the concentration of the subunit Cdc23 (present in two copies in APC/C) using FCS [4]. We measured it as ~ 30-35 nM.

In the model we set A_{total} to 40 nM, which corresponds to 100 molecules/cell (see later for the translation from concentration into protein numbers).

Total Mad (Mad_{total}): Mad2 (~200 nM) and Mad3 (~70 nM) were also measured by FCS [4]. Since Mad3 is limiting, in the model we set the Mad variable to 70 nM (175 molecules/cell).

Fitted parameters

For the parameters that could not be measured directly, we set an initial value which was similar to the model developed in [4] and bistable at the same time; then they were optimized by Sloppycell in order to fit experimental data.

Experimental results used for the fitting

For fitting parameters with Sloppycell, we used the following experimental results as input data: (i) concentrations of proteins and complexes at steady state; (ii) comparison of background vs APC/MCC-catalyzed Cdc20 degradation; (iii) bistability.

(i) Concentrations at steady state

We looked for parameters that would satisfy specific concentrations at steady state with an active checkpoint. Here we describe for all such concentrations the value measured experimentally and the value given for the fitting algorithm. The algorithm assigned parameters to fit these data, accepting concentrations in a region around the given value. The final concentrations in the model and the parameters are described in Tables 1 and Table 2.

Total Cdc20 (i.e. free Cdc20 as well as all complexes containing Cdc20): This quantity is not conserved since Cdc20 is constantly synthesized and degraded. By western blotting, we can see that Cdc20 and APC subunits are present in similar amounts during checkpoint arrest (Fig 2E [4]). Hence, for Cdc20 at steady state we used the same value reported for APC/C : ~ 30-35 nM (set to 100 molecules/cells for the fitting).

APC/CMCC was measured by FCCS to be ~10 nM (we set ACMC to ~30 molecules/cell) (Figure 4A [4]).

Total MCC (i.e. the combination of **MCC** *and* **APC/CMCC)** was measured by FCCS to be ~13 nM (~35 molecules/cell) (Figure 4B [4]).

Indirectly, we inferred steady state concentrations of all other species:

We obtain MCC (MC in the model) by subtracting APC/CMCC from total MCC, which gives ~ 3 nM. In the model we used 5 molecules/cell for MC.

APC+APC/Cdc20 = APCtotal-APC/CMCC= ~ 70 molecules. Without further supporting data, we used ~40 molecules/cell for A and 30 molecules/cell for AC with a larger *acceptance region*, and we let the fitting algorithm find an optimal value.

For free Cdc20 (C in the model) we used a value of 5 molecules/cell, calculated from [Cdc20 total - (APC/Cdc20 +2*APC/CMCC + MCC)]. The factor 2 takes into account that APC/CMCC contains two molecules of Cdc20.

For **Mps1**, we do not have any measurement, so we introduced a loose constraint that it should have a small value around 40 molecules/cell. Such a small value is in line with the kinase not being detectable when GFP-tagged [5].

In Figure M1, we show the concentrations of fitted values for steady states during the checkpoint ON state compared to the experimental values.

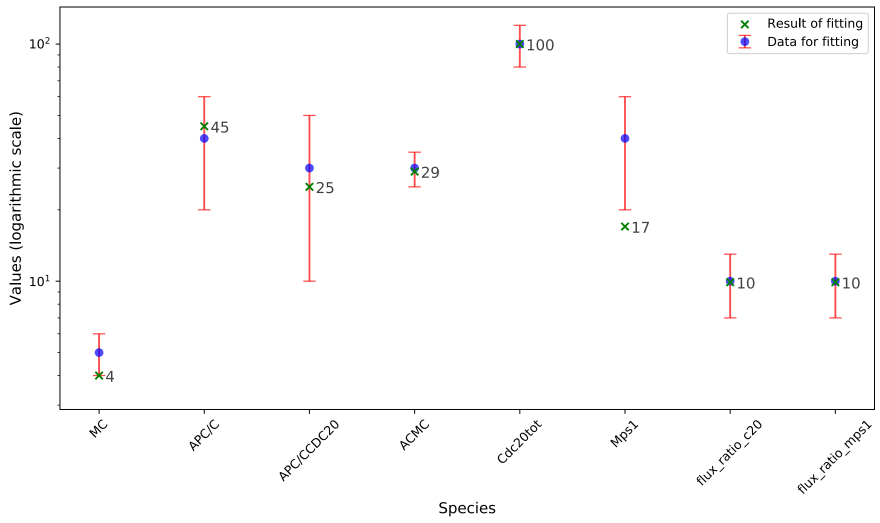

Figure M1. Comparison of fitted and experimental values. In the plot we compare data used for fitting and the results of the fitting done by sloppycell. Red bars show the range of accepted values, blue dots are the initial suggested data and green crosses the fitted values.

(ii) Comparison of background vs APC/MCC-catalyzed Cdc20 degradation

As a second constraint in the parameter fitting we assumed that Cdc20 background degradation should be much lower than the well-documented APC/C-dependent degradation. We included background degradation to avoid unrealistically high concentrations in the checkpoint OFF state, but we did not want it to have a significant impact on the checkpoint ON state. Hence, we asked the rate of Cdc20 degradation via the background path to be at least 10 times smaller than the rate of degradation via APC/CMCC.

(iii) Bistability

Finally, we asked the algorithm to search for parameter values that would give bistability for 10 unattached kinetochores. This was achieved by asking sloppycell to identify a checkpoint OFF steady state where all checkpoint species were fully inactive.

Parameters for simulation of the mutants

APC-A mutant:

Using FCCS, it was observed that APC-A produces as much APC/MCC as the wild type, but more MCC.: **APC/CMCC** was measured by FCCS to be ~10 nM (~25 molecules/cell), while total **MCC** was measured to be ~16 nM (~40 molecules/cell) (Figure S4B)

In the model we introduced a 35% decrease in k_{assAC}, the association rate constant of AC-binding. At steady state this resulted in 29 molecules/cell of $ACMC$ and $ACMC + MC$ to be 36 molecules/cell in good agreement with the data.

Manually, we also asked for a marked reduction of adaptation events when the number of unattached kinetochores is fixed at 10.

GM2 mutant: For simulating Mad2 overexpression with the model, we increased the total amount of Mad so that it matches the decrease in adaptation events under constant drug arrest in the mutant (by 600 minutes 20% of cells adapt) Figure S5A.

As a result Mad_{total} was set to 220 molecules/cell.

Additional parameter determination

Fitting was done on the deterministic model using Sloppycell. Satisfactory parameter sets were then manually tested with stochastic simulations for adaptation dynamics keeping constant the number of unattached kinetochores (Figure 2B-2E).

Finally, the parameters for checkpoint signal at the kinetochores ($J_n$and k') were adjusted so that the saddle node lies between 0 and 1.

Methods for the deterministic model

Deterministic ordinary differential equation model and bifurcation analysis were done using the software XPPAUT [6].

For deterministic simulations we used two numerical integrators: the Runge-Kutta and the CVODE method.

In every simulation we set parameter boundaries to 1000 and integration time to 2000 time units.
In case of CVODE method we used the following settings: relative tolerance = 0.0001, absolute tolerance = 0.0001.

Once a steady state of the system we used AUTO for bifurcation analysis to track the solution of the system as one parameter is varied

AUTO was used with the following settings:

@ dsmax=.5, dsmin=.0001, ds=-.02, ntst=1500, nmax=2000, npr=500, parmin=0, parmax=32
@ autoxmin=0, autoxmax=1, autoymin=0, autoymax=100

The ODE model was converted into a SBML (Systems Biology Markup Language) model of biochemical reactions that can be used for simulations in Python. The Python package called 'SloppyCell' was used to fit the total amount of proteins and the concentrations of the protein complexes to experimental values.

Stochastic model

Stochastic simulations were performed with the Python package 'StochPy' using Gillespie's direct method [7].

Parameters

In stochastic simulations we transformed concentrations into molecules/cell. Importantly, all reactions of interest take place in the nucleus and not in the whole cell. The conversion factor was calculated using the mean nuclear radius of a haploid yeast cell of ~ 1µm. This radius corresponds to ~ 4.2 μm^3 nuclear volume using the approximation that yeast nuclear volume corresponds to the volume of a sphere. Concentrations were measured in nanomolar (nM). From the nuclear volume and the Avogadro number, we obtain for the molecule number / molar concentration ratio $2.5 \cdot 10^6\ m^3$ /mol. The conversion factor is thus ~ 2.5 molecules/nM .

Transcriptional burst noise:

In the stochastic model we introduced an additional source of noise originating from transcriptional bursting of the two species that were synthesized and degraded: Cdc20 and X. The bursting was modeled assuming that their promoter can exist in two distinct states: a transcriptionally active ON state and an inactive OFF state following [8]. The promoter switches stochastically between these states with rates k_on and k_off.

Simple Models

We developed two simple models, one based on Figure 1A left (model 1), and one on Figure 1A right (model 2).

Simple model 1

The deterministic version of the first simple model can be translated to the following two differential equations: APC can be present in two forms, either active or inactive (calculated as the total – the active form). It is inactivated by species X and unattached kinetochores (nuk). Additionally, the Hill equation was used to introduce nonlinearity in the system.

$$d\frac{APC_a}{dt} = ka \cdot (APC_{tot} - APC_a) - ki \cdot X \cdot APC_a \cdot nuk$$

X is synthesized and degraded by active APC. We introduced an additional background degradation besides the APC mediated primary degradation to avoid unrealistically high concentrations in the checkpoint OFF state.

$$d\frac{X}{dt} = ks - kdeg \cdot \frac{APC_a^n}{J^n + APCa^n} \cdot X - kdeg_{BG} \cdot X$$

We used the following arbitrary parameters:

Description	Parameter	Value (deterministic)	Value (stochastic)
APC activation rate	k_a	$0.3\ min^{-1}$	$0.3\ min^{-1}$

APC inactivation rate	k_i	$0.08\ (nM\ \cdot min)^{-1}$	$0.2\ (mol\ \cdot min)^{-1}$
X synthesis rate	k_s	$0.08\ min^{-1}$	$0.08\ min^{-1}$
X degradation rate	k_{deg}	$0.24\ (nM\ \cdot min)^{-1}$	$0.6\ (mol\ \cdot min)^{-1}$
X background degradation	k_{degBG}	$0.008\ min^{-1}$	$0.02\ min^{-1}$
MM constant	J	4 ?	10 ?
Hill coefficient	n	3	3
Total APC	APC_{tot}	$40\ nM$	$100\ mol./cell$

Parameters modified for simulations

For the simulation which served as a proof of bistability (Figure S6B-S6C) in this simple model we introduced an overexpression of X, by increasing the synthesis rate of X (k_s) by 3-fold.

For simulating the APC-A mutant we decreased to half the activation rate of APC (k_a).

Simple model 2

The model with positive feedback loop on APC activation is one dimensional; here X does not play any role in the positive feedback loop and is neglected from the equations.

APC can be present in two forms, either active or inactive (calculated as the total – the active form). Active APC helps its own activation. It is inactivated by unattached kinetochores (nuk). Additionally, the Hill equation was used to introduce nonlinearity in the system.

$$d\frac{APC_a}{dt} = (APC_{tot} - APC_a) \cdot \left(k_a' + k_a \cdot \frac{APC_a^n}{J^n + APC_a^n}\right) - ki \cdot APC_a \cdot nuk$$

We used the following arbitrary parameters:

Description	Parameter	Value (deterministic)	Value (stochastic)
APC self- activation rate	k_a	$1\ min^{-1}$	$1\ min^{-1}$
APC inactivation rate	k_i	$0.3\ min^{-1}$	$0.3\ min^{-1}$
APC activation rate	k_a'	$0.005\ min^{-1}$	$0.005\ min^{-1}$
MM constant??	J	27.5 ?	27.5 ?
Hill coefficient	n	3	3
Total APC	APC_{tot}	$40\ nM$	$100\ mol./cell$

Parameters modified for simulations

For the simulation which served as a proof of bistability (Figure S6H-S6I) in this simple model we simulated an overexpression of X indirectly, by increasing the inactivation rate (k_i) by 3-fold.

For simulating the APC-A mutant we decreased to half the activation rate of APC (k_a).

Tables

1. Aravamudhan, P., Chen, R., Roy, B., Sim, J., and Joglekar, A.P. (2016). Dual mechanisms regulate the recruitment of spindle assembly checkpoint proteins to the budding yeast kinetochore. Mol Biol Cell *27*, 3405-3417.
2. Gutenkunst, R.N., Atlas, J.C., Casey, F.P., Daniels, B.C., Kuczenski, R.S., Waterfall, J.J., Myers, C.R., and Sethna, J.P. (2007). SloppyCell. (http://sloppycell.sourceforge.net).
3. Myers, C.R., Gutenkunst, R.N., and P., S.J. (2007). Python unleashed on systems biology. Comput Sci Eng *9*, 34-37.
4. Bonaiuti, P., Chiroli, E., Gross, F., Corno, A., Vernieri, C., Stefl, M., Cosentino Lagomarsino, M., Knop, M., and Ciliberto, A. (2018). Cells Escape an Operational Mitotic Checkpoint through a Stochastic Process. Curr Biol *28*, 28-37 e27.
5. Huh, W.K., Falvo, J.V., Gerke, L.C., Carroll, A.S., Howson, R.W., Weissman, J.S., and O'Shea, E.K. (2003). Global analysis of protein localization in budding yeast. Nature *425*, 686-691.
6. Ermentrout, B. (2002). Simulating, Analyzing, and Animating Dynamical Systems: A Guide to XPPAUT for Researchers and Students, (SIAM).
7. Maarleveld, T.R., Olivier, B.G., and Bruggeman, F.J. (2013). StochPy: a comprehensive, user-friendly tool for simulating stochastic biological processes. PLoS One *8*, e79345.
8. Lammers, N.C., Kim, Y.J., Zhao, J., and Garcia, H.G. (2020). A matter of time: Using dynamics and theory to uncover mechanisms of transcriptional bursting. Curr Opin Cell Biol *67*, 147-157.
