# Supplementary material for: lncreased risk of slippage upon disengagement of the mitotic checkpoint
Source: PLoS Comput Biol. 2025 Mar 19;21(3):e1012879. doi: 10.1371/journal.pcbi.1012879 (PMC11981154; doi:10.1371/journal.pcbi.1012879)
Supplement: Attachment — Submitted filename: 20250210_answer_rev_PLoSCB.pdf [file pcbi.1012879.s013.pdf]

Response to Reviewers - Stier et al.

Reviewer #1:

In this paper, Stier et al. study the phenomenon of adaptation to ('slippage from') the spindle assembly checkpoint (SAC) in budding yeast cells, by a combination of judicious experiments and careful computational modeling. In my opinion, the experiments and modeling are done very professionally, and they provide new insights into the mechanisms whereby yeast cells bypass the SAC and exit mitosis with chromosomes that are not properly aligned on the mitotic spindle. This is an important problem in cell biology, with implications for carcinogenesis in human cells. I recommend the paper for publication in PLOS Comp Biol after minor revisions.

I have a few comments and suggestions that should be addressed in a revision:

We thank the Reviewer for appreciating our work.

1. In the Abstract, the authors say, "According to the model, drug removal further facilitates adaptation, a prediction we confirmed in budding yeast." That is true: the computational model predicts that removing the checkpoint signal (nocodazole) causes more cells to enter anaphase with unaligned chromosomes (adaptation) than would happen with the checkpoint in place, and the authors confirmed this prediction. But, on p 9, when introducing these simulations and experiments, the authors say, "We reasoned that dismantling the checkpoint by washing out nocodazole may achieve this goal" (i.e., the goal of "entering anaphase in the presence of unattached kinetochores"). They don't explain their reasoning. Why is it counterintuitive to think that, once the checkpoint is engaged by nocodazole and many chromosomes are unattached to the spindle, removing the checkpoint inducer would increase the probability of slippage? What causes them to think removing the signal would decrease slippage? In the following paragraph, they say, "In contrast to expectation (i.e., to their reasoning), the majority of cells (60.8%) started Clb2 degradation in the presence of localized Mad2 even after washout." Should we be surprised? Their model simulations (Fig. S3C) don't help: during normal exit, fluctuations don't flip the checkpoint OFF until nearly all the kinetochores are aligned, but during adaptation, fluctuations flip the checkpoint OFF with many (or a few) unaligned kinetochores. What's the difference in these simulations? What do they have to do with removing nocodazole? Since these experiments and simulations are a major conclusion of the paper, they must be explained more clearly.

Our reasoning was that, after wash out, one could have maybe avoided adaptation if microtubules had stabilized before cells adapted. If microtubules had polymerized and connected to kinetochores before adaptation took place, washing out would have avoided adaptation. We agree that the argument is confusing, and so we rephrased the flow of our reasoning. Now we introduce the washout simply asking what would happen removing the drug, since this is a condition closer to what happens during actual treatment, when the drug is given in shots.

The reviewer then asks us about the simulations in Figure S3C. Here, we show trajectories of identical cells which have different fates entirely by chance, driven by noise. We suspect that a source of confusion was our definition of 'regular exit'. We only perform simulations starting from steady state conditions (i.e., full engagement of the mitotic checkpoint). In this context, we understand that the term 'regular exit' is misleading, since it naturally suggests a normal cell cycle, which we never simulate. This would have required a formal description of the additional restraints on APC/C activation that the reviewer mentions in the next point: APC/C phosphorylation by Cdk1/CyclinB to allow Cdc20 binding, Cdc20 accumulation, etc. What we meant with 'regular exit' is rather an exit from metaphase that takes place when the SAC is satisfied (i.e., silenced) because all kinetochores have been attached. For the sake of clarity, we rephrased our terminology throughout the manuscript and in the Figures, replacing 'regular exit' with 'checkpoint satisfaction'. Hopefully with these changes Figure S3C becomes clearer.

2. I also have some issues with the discussion of the "steady-state trap". It is true that, during normal cell cycles (in absence of nocodazole), the frequency of mis-segregation is very low, but in response to nocodazole treatment (sustained or temporary), the frequency of mis-segregation is high. Furthermore, during normal cycling, the SAC genes are dispensable, presumably because there is plenty of time for chromosomes to be properly aligned on the spindle before other restraints on APC/C activation are lifted. As the authors say, "the mitotic checkpoint is only transiently, if at all, activated during a regular yeast cell cycle." The checkpoint mechanism, once engaged, "stalls cell division, but it struggles to keep APC/CCdc20 levels under control." That is true, since spontaneous fluctuations may kick a cell out of the domain of attraction of the checkpoint ON state. Whether or not adaptation occurs depends on the height of the barrier (about 20 molec/cell in Fig. S3C) and the number of molec of APC/CCdc20 in the ON state (~25 in Fig. S3C). If fluctuations are Poisson, then their std dev is 5 molec and an excursion of 20 molec should be quite rare; correct? Are these the fluctuations we are seeing in Fig. S3C?

I suspect that "adaptation" has a selective advantage in yeast cells. If, for any reason, a yeast cell gets stalled in mitosis by the SAC, its best strategy may be to slip out of the checkpoint and "take its chances" to produce viable progeny rather than to remain stuck in mitosis. Of course, in multicellular organism, such slippage can be catastrophic (carcinogenic).

The reviewer is correct: cells can escape from the trap only with consistent fluctuations. And it is correct that fluctuations of the size observed in our simulations would be rare for purely

intrinsic noise due to the small number of molecules. However, in our model we additionally introduced noise due to transcriptional bursts in protein synthesis that are well documented in yeast (Lammers et al, Curr Op Cell Biol, 2020 -- PMID 33242838) and lead to higher fluctuations. We tried to clarify the point in the revised manuscript ("Furthermore, we developed a stochastic version of the model to investigate whether random fluctuations could enable spontaneous transitions between the steady states. We found that the intrinsic noise supplemented with transcriptional bursts, which are well documented in yeast [19], suffice to produce spontaneous transitions from the checkpoint ON to the checkpoint OFF state (but not vice versa).").

Finally, we agree with the evolutionary interpretation of adaptation, and we thank the reviewer for this suggestion. We introduced this argument in the discussion, when comparing yeast and mammalian cells.

3. Table 1: the authors mean molec/nucleus rather than molec/cell.

The reviewer is correct, budding yeast undergoes a closed mitosis where all these reaction take place. We change the caption of Table 1 accordingly.

4. Table 2: why are some parameter values specified to 6 significant figures (16.9063 nM/min) and others to only one (0.03 /min)?

The reviewer is correct, we were not consistent. Parameters produced by the fitting software were given with 6 significant figures, and so with much higher precision than parameters set manually. In the revised version we report parameters in a consistent way with only two significant figures. The exact values used for the simulations can be found in the source files, now available on github (see point 7). To double check that this is not misleading, we performed a simulation using 2 significant figures for all parameters, and we obtained basically the same bifurcation diagram (below, the two plots fully overlap). To confirm that the stochastic simulations also do not change, we reproduced Figure 2E (cumulative distribution of adaptation time in the constant presence of the drug). This result summarizes fundamental dynamical properties such as bistability and likelihood of transition. Reassuringly, also the cumulative distribution of adaptation times was consistent, as shown below.

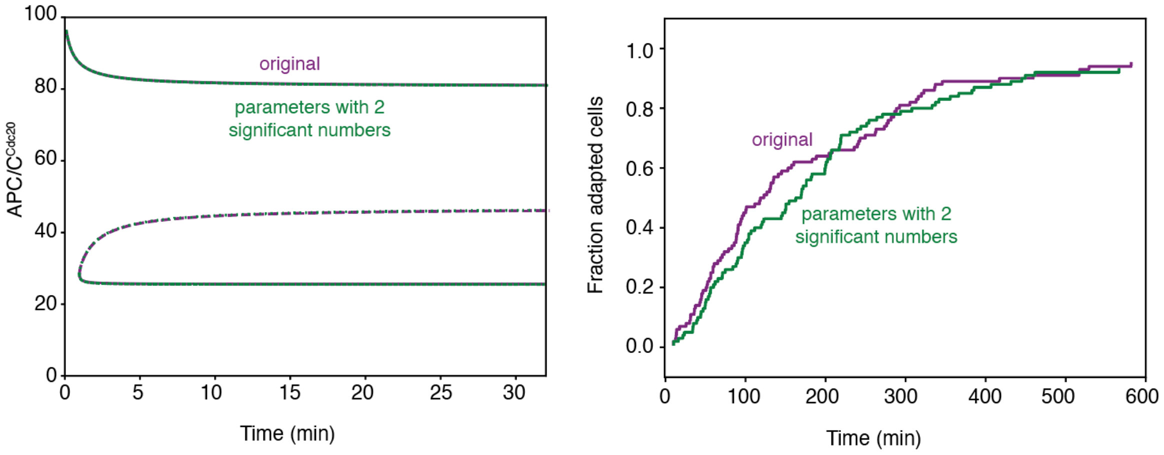

5. Table 3: Madi → Mada rate is described by Michaelis-Menten kinetics, but MM kinetics is notoriously suspect in stochastic calculations, as the senior author knows well. A word of explanation may be in order.

The reviewer is right: our model is entirely mass-action, except for Mad2 activation, which is described by a couple of Michaelis-Menten reactions. These add non-linearity in the system, but their introduction in large networks should be treated with care. The MM is simplified ('packed') thanks to quasi-steady state approximations, which may alter qualitatively the behavior of molecular networks, up to the 'invention' of multiple steady states. This is even more true when one couples together multiple michaelis-menten reactions, as we did. Alternative approximations (like the total quasi-steady state approximation, tQSSA) are better suited for larger networks. In other words, the reviewer is asking whether in the deterministic model the QSSA invents new steady states; in the stochastic model, whether simulations are still accurate.

To address these problems, first we tested whether in fact both michaelis-menten approximations are needed. It turned out this is not the case: we can have a simple linear term for Mad inactivation (Mad1i -> Mad2a) without losing bistability, in fact we obtain the same bifurcation diagram (full overlapping in the plot below). With such a model, the cumulative distribution of adaptation times also overlaps the one included in the model. This makes one of our assumptions less 'dangerous'.

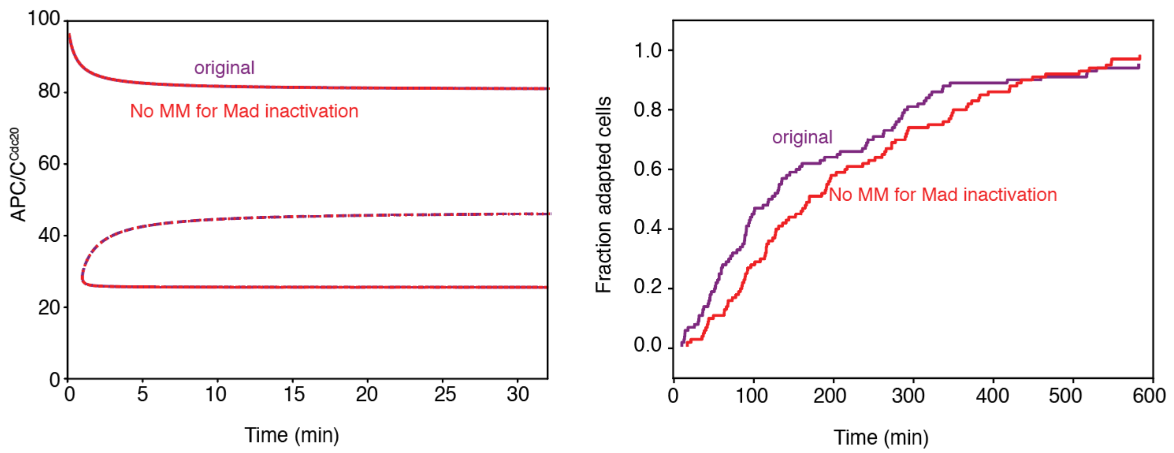

Second, we asked whether a purely mass-action model would also show bistability. Provided such a model can be then simplified to obtain an equation for Mad2 activation similar to what was used in our simulations, we could show that using the michaelis-menten approximation does not create artificial qualitative behaviors. In fact, this exercise can be relevant also to address the other steady state approximations that implicitly we use for checkpoint components activation and binding to kinetochores.

Hence, to show that the approximations do not alter consistently the model's behavior, we aimed at: (i) identifying a mass-action model for Mad2 activation that shows bistability when plugged in the larger model ('unpacked model'); ii) showing that it can be simplified ('packed model'), while maintaining the properties of the unpacked model; and (iii) showing that equations, bifurcation diagram and stochastic simulations of this packed model are similar to those of our 'original model'.

A mass-action model for Mad2 activation

In the original model used in the paper, the module of Mad2 activation is described by this equation:

$$\frac{dM^*}{dt} = k_{act}\frac{M}{M+J}\frac{Ut}{Ut+Kd} \cdot Mps1 - k_{inact} \cdot M^* \qquad (1)$$

Here, M is inactive Mad2, M* is active Mad2, Ut is the total number of unattached kinetochores (nUK in the paper), J is the Michaelis Menten constant for M* production, Kd is the dissociation constant for the binding of SAC components to unattached kinetochores, kact is a rate constant which accounts for the catalytic activity of kinetochore/checkpoint components. Mps1 contributes to activate the checkpoint.

One possible mass-action version of these reactions has three layers: (i) priming of checkpoint components (we call them S, from the checkpoint acronym 'spindle assembly checkpoint' or SAC) by Mps1, (ii) binding of primed SAC components to unattached kinetochores; (iii) bound checkpoint components catalyze the formation of active Mad2. The

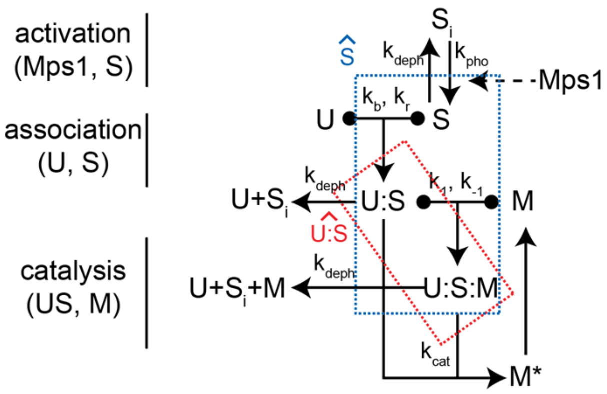

wiring diagram for this network is shown on the left: U represents unattached kinetochores; S represents generic SAC components other than Mad, which have been shown to be limiting (i.e., not all binding sites on kinetochores are occupied by SAC components (Aravamudhan et al, MBC, 2016, PMID: 27170178); Mps1 determines the amount of SAC components that can bind to kinetochores.

This network is described by the following algebraic-differential equations:

$$U = Ut - \widehat{U:S} \tag{2}$$

$$S_i = St - \hat{S} \tag{3}$$

$$S = \hat{S} - \widehat{U:S} \tag{4}$$

$$U:S = \widehat{U:S} - U:S:M \tag{5}$$

$$M^* = Mt - U:S:M \; - \; M \tag{6}$$

$$\frac{dM}{dt} = -k_{cat} \cdot U:S:M + k_{inact} \cdot M^* \tag{7}$$

$$\frac{d\widehat{U:S}}{dt} = k_b \cdot U \cdot S - k_r \cdot U:S \; - k_{deph} \cdot \widehat{U:S} \tag{8}$$

$$\frac{dU:S:M}{dt} = k_1 \cdot U:S \cdot M - U:S:M \cdot (k_{-1} + k_{cat} - k_{deph}) \tag{9}$$

$$\frac{d\hat{S}}{dt} = k_{pho} \cdot S_i \cdot Mps1 - k_{deph} \cdot \hat{S} \tag{10}$$

Full unpacked model

To study the behavior of the mass-action Mad2 activation module in the complete network, we plugged the module described previously in the complete wiring diagram.

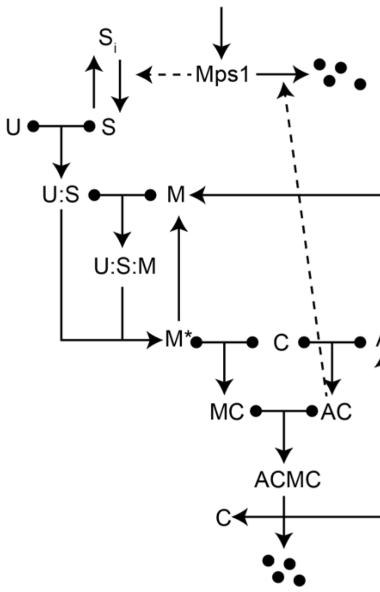

We then performed bifurcation analysis, and observed bistability keeping unaltered all parameters present in the original model. These results are shown in the Figure here below. Noticeably, we notice that bistability is not lost, and the stochastic transitions in the presence of unattached kinetochores is quite similar to the one produced by the model used in the paper. The unpacked.ode file can be found at the end of the rebuttal.

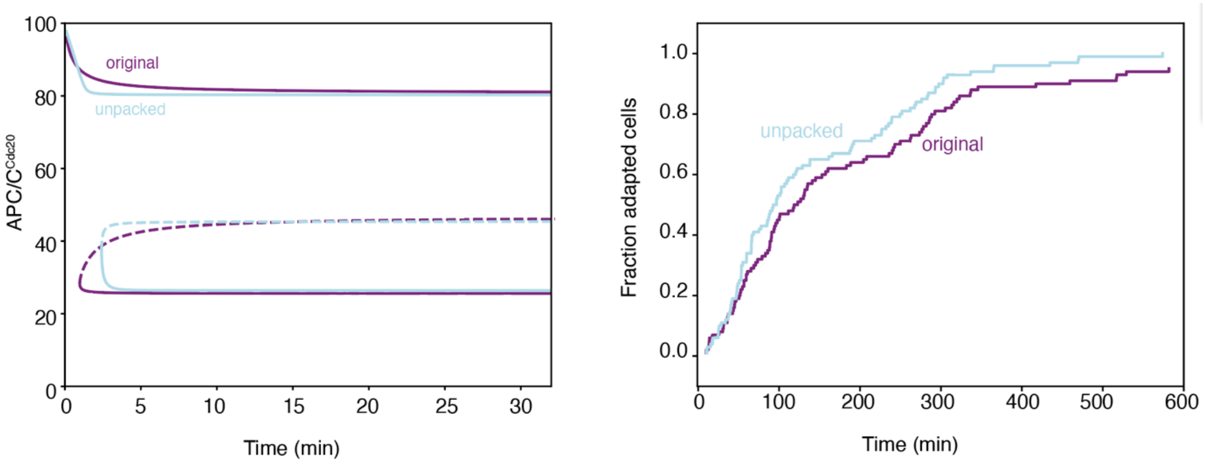

Steady state approximations of the Mad2 activation module: packed model

We then set out to show that this mass-action model, when simplified with steady state assumptions for all species but Mad2 (i.e., M and M*), can give equations similar to those we used in the manuscript. First, we analyzed the isolated Mad2 activation module. If Sac activation/inactivation are fast (equation 10 is set to zero), the total amount of Sac components primed for kinetochore binding can be calculated as:

$$\hat{S} = St\frac{Mps1}{Kph+Mps1} \qquad (11)$$

where

$$Kph = \frac{k_{deph}}{k_{pho}}$$

Equation (8) instead, assuming fast binding/dissociation and using (2) and (11), would give as steady state solution:

$$\frac{d\widehat{U:S}}{dt} = k_b \cdot (Ut - \widehat{U:S}) \cdot (St \frac{Mps1}{Kph+Mps1} - \widehat{U:S}) - k_r \cdot U:S \; - k_{deph} \cdot \widehat{U:S} = 0 \qquad (12)$$

Since the number of checkpoint protein capable to bind kinetochores is limiting, we can assume $Ut >> St \frac{Mps1}{Kph+Mps1}$, and we get

$$0 = k_b \cdot Ut \cdot (St \frac{Mps1}{Kph+Mps1} - \widehat{U:S}) - k_r \cdot U:S \; - k_{deph} \cdot \widehat{U:S} \qquad (13)$$

Solving (9) using definition (5) we obtain

$$U:S:M = \frac{\widehat{U:S} \cdot M}{M+J} \qquad (14)$$

where $J = \frac{k_{-1}+k_{cat}+k_{deph}}{k_1}$ is a modified michaelis-menten constant.

replacing (5) and (14) in (13) we find that

$$\widehat{U:S} = \frac{Ut \cdot St \cdot \frac{Mps1}{Mps1+K_{ph}}}{Ut+K_D\frac{J}{J+M}+K} \;, \qquad (15)$$

where $K_D = \frac{k_r}{k_b}$ and $K = \frac{k_{deph}}{k_b}$

Hence, we find from (7), (14) and (15):

$$\frac{dM}{dt} = -k_{cat} \frac{M}{M+J} \cdot \frac{Ut}{Ut+K_D\frac{J}{J+M}+K} \cdot \frac{Mps1 \cdot St}{Mps1+K_{ph}} + k_{inact} \cdot M^* \qquad (16)$$

Equation (16) is quite similar to equation (1) which we used in the original model, with Mps1 being 'weighted' by the total amount of SAC components St (i.e., the fraction of S capable to interact with kinetochores). By choosing a Kph large enough and adjusting the value of J, when we replace equation (1) with equation (16) we obtain a bifurcation diagram similar to that produced by our original model. Not only the deterministic behavior is similar, but also the cumulative distribution of adaptation times produced with stochastic simulations is very similar to that produced by the original model as shown below. The packed.ode file can be found at the end of the rebuttal.

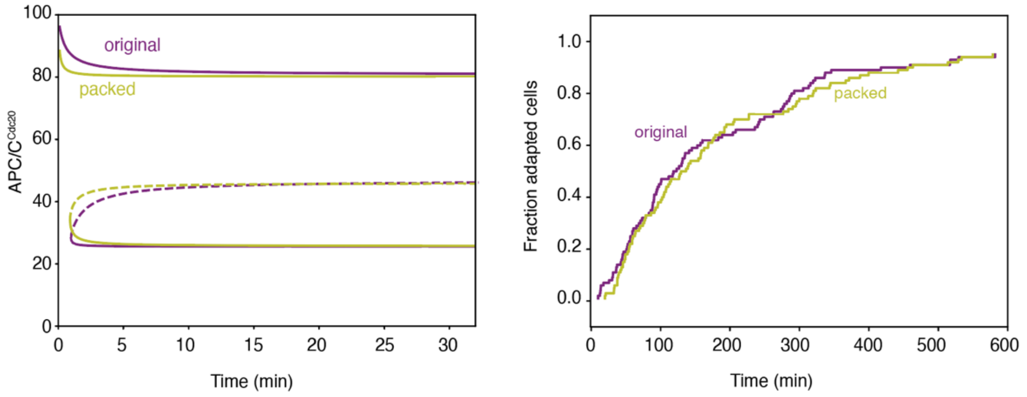

In summary, we showed that bistability in the same region observed for the original model could be obtained by a full mass-action model which includes explicit binding of Mad2 together with additional SAC molecules to kinetochores. The approximated form assuming steady state solutions for the reactions leading to Mad2 activation is similar in equation, bifurcation diagram and stochastic simulations to our original model and thereby validates it.

Given the similar results of our original model and the new 'packed' model, we did not repeat all the fitting and simulations with this latter, but we kept the results obtained with the original model. This is also in the spirit of the paper, where we show that the behavior we observe does not depend strictly on the precise equations, but it is rather a generic property of positive feedback loops. Similarly, we consider the stochastic simulations to be satisfactory if they produce the pattern of transition times that we observe experimentally. We do not have data to quantitatively study the behavior of the stochastic trajectories of the system, and therefore consider it neither possible nor necessary to have a model that is validated at this level of precision.

6. The authors are inconsistent in notation: sometimes APC/CCdc20 and sometimes APC/C^{Cdc20}*. I prefer the latter, or APC/C-Cdc20. But be consistent.*

Now the text is consistent; a similar notation was used for MCC (i.e., APC/C^{MCC}).

7. The authors should make all computer code publicly available.

The files for deterministic and stochastic simulations are available at https://github.com/sbeatrix/WIA_source_codes/tree/main

We included with the revision the files used for the fully unpacked and new packed models described above, although we do not plan to include in the final version of the paper.

Reviewer #2: In their article, Stier, Bonaiuti et al describe a mathematical model that models the mitotic checkpoint in yeast and gives a rationale how cells exit the checkpoint after prolonged misattachment of microtubli.
The model is a network model, is build upon a previous model where they added components for the checkpoint "sensor" and consists of a positive feedback and is formulated as ordinary differential equations. A deterministic analysis shows that the dynamics is bistable. A stochastic analysis of the model shows an escape kinetics that is similar to the kinetics observed in experiments (Fig 2B+E), although there is some difference (a delay in the

experimental data.
They test the model with specific experiments. For instance, they compare how many cells escape the checkpoint if the checkpoint is only temporarily activated, or they perform overexpression experiments to proof the bistability experimetnally.

Overall I am very enthusiastic about this paper. It is a carfuly created model for a very relevant process that is very well supported by experimental data and really answers the question why cells escape the mitotic checkpoint. The experiments have been well thought through and I am particularly enthusiastic how the authors provide experimental evidence for bistability.

We thank the reviewer for judging so favourably our work.

I have only very minor comments:

1) I am curious how the authors explain the delay in escape in experimental data (Fig. 2B). I assume that if you need to overcome a bareer in a bistable system by stochastic fluctuations, this would result in an exponental escape distribution (something similar you see in Fig. 2E). Is there a delay in when escape can be detected?

The reviewer is correct. This is a relevant difference we did not explain. The plot in Figure 2B shows the experimental cumulative distribution of mitotic arrest time, defined as the time between the rise and the fall of Clb2 levels, as detected by the Clb2-mCherry signal. The delay in this plot shows that in real cells something needs to happen before Clb2 can be degraded. This something is the formation of the APC/C^{Cdc20} complex, which requires both APC/C phosphorylation and Cdc20 production, both driven by Clb2-CDK1 activity itself. Hence, APC^{Cdc20} activation cannot start immediately after Clb2 increases. In other words, the experimental system is not yet at steady state.

In the simulations (Fig 2E), by contrast, we start from the steady state solution where APC/C^{Cdc20} is already formed, and the transient is missing. Hence, the distribution is purely exponential: fluctuations can immediately lead to APC/C^{Cdc20}-driven Clb2 degradation . In the experimental system, instead, it is exponential after a delay. We clarified this point in the text.

2) In parts the experimens are difficult to follow for compuational people. For instance, the nocodazole washout experiment would not be understandable for people who don't know the compound. Please go through the article and explain better what these experiments test and what the molecular consequence of e.g. nocodazole washout is.

We changed the introduction, to explain more clearly how microtubule-targeting drugs arrest cell cycle progression. We also added extra explanations every time we performed an experiment to better clarify its meaning and technicalities. We hope in the revised form it is more understandable by computational scientists.

Reviewer #3: Review of Ciliberto 2024

Disclaimer: I am not qualified to assess the computational model, so my comments are focused on the experimental sections.

This study investigates the mechanisms underlying mitotic checkpoint adaptation (or "slippage") and its relationship to chromosome missegregation and aneuploidy. Using a combination of mathematical modelling and experimental validation in budding yeast, the authors demonstrate first, that slippage is stochastic, driven by fluctuations in APC/C-Cdc20 activity, and second, that chromosome missegregation is exacerbated during or after nocodazole washout. The paper identifies two potential interventions to mitigate slippage by weakening the checkpoint -- with APC mutants or Mad2 overexpression. These insights may be relevant for enhancing the efficacy of microtubule-targeting drugs in therapeutic contexts. However, comparisons with mammalian cells are difficult given important differences between the two systems.

The study combines predictive modelling with detailed experimental validation, which is a powerful combination that strengthens the robustness of its findings. The paper introduces a bistability-driven framework for understanding mitotic checkpoint adaptation, providing a new perspective on mechanisms generating aneuploidy. In particular, the observation that nocodazole washout significantly increases the likelihood of chromosome missegregation and aneuploidy is particularly compelling, and in my opinion could be given more weight.

I recommend addressing the following issues that may significantly improve the paper before publication.

We thank the reviewer for appreciating our work.

1. My main criticism is that I found the manuscript's results difficult to follow because related experiments are dispersed across multiple figures (e.g., wild-type and mutant data for adaptation are split between Figures 3A, 4B, and 5D). Consolidating these into one or two cohesive figure(s) or section would clarify the message and improve readability.

We understand the reviewer's point of view. From the perspective of an experimental biologist, comparing all the experimental data would come more naturally. But here, we thought it was more relevant to compare simulations and experiments. We tried different versions of the paper to address the reviewer's concern, but we always found the final result unsatisfactory in terms of combining experiments and simulations. Hence, although we understand the point of the reviewer, we decided to maintain the Figure organization that we had originally proposed.

2. The finding that cells are significantly more prone to chromosome missegregation after nocodazole washout seems to me like a novel and important insight. This result, shown in Figure 3A and related panels in other figures, should be highlighted more prominently, as it provides a clear link between checkpoint adaptation and aneuploidy. It should also be documented more clearly. For example, it would be nice to include in the main figure images of representative cells undergoing exit and adaptation, and mention the protocol for nocodazole washout (using microfluidics) in the main text.

We explained in more details the protocol with microfluidics in the main text. We also produced two movies with examples of satisfaction and adaptation, which are now available as supplementary movies. Finally, in the revised text we remarked the importance of our

findings both for the synchronization experiments performed using nocodazole, and for the potential implications in terms of treatment with microtubule-targeting drugs.

3. Related to this, Figure 3A shows that 60% of cells adapt upon nocodazole washout, based on the observation that Clb2 degradation starts in the presence of Mad2 clusters, which should correspond to unattached kinetochores. Moreover, the model predicts that the time of adaptation for each cell is inversely correlated with its number of unattached kinetochores. However, this correlation is much lower for experiments than for the models, so I find the use of Mad2 intensity in Figure 3C as a proxy for unattached kinetochores unconvincing. Is the correlation driven mainly by the three data points with high Clb2 degradation times in graph 3C right? Much more compelling evidence for missegregation during adaptation is provided for chromosome V in Figures 5E and S5G, but requires confirmation that protocols are consistent across these experiments.

The reviewer's comment addresses the connection between missegregation and adaptation. Adaptation is chromosome segregation without proper microtubule-chromosome attachment. As such, it is predicted to result in missegregation. However, cause-effect relationship in quantitative terms is hard to find in the literature. Following the reviewer's suggestion, we addressed this point in different ways:

- We clarified in the text that the protocol for studying missegregation of chr V (Figure 5E and S5G) is identical to the protocol used for studying adaptation (Figure 3A and Figure 4B).

- Based on that, we connected quantitatively adaptation and missegregation for APC-A and WT. Not only we found that they are correlated, but assuming that all chromosomes share the probability of missegregation, we find that the fraction of adapting cells goes with the fraction of cells that missegregate at least one chromosomes. In other words, if cells adapt, they are likely to missegregate at least one chromosome. The results are shown in the new Figure S5H, shown below:

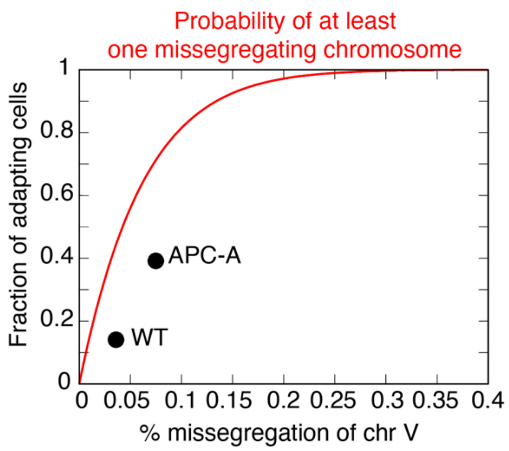

This conclusion, based on the two mutants for which we could measure both missegregation and adaptation (i.e., APC-A and WT), is new and indeed very interesting. We thank the reviewer for having suggested us to further investigate in this direction.

- Finally, the reviewer asks whether Mad2 localization is a good proxy for unattached kinetochores, pointing at the 3 specific points with late Clb2 degradation. We acknowledge that the visualization we chose was not the best. The accumulation of many points in a limited space hindered the possibility of appreciating the correlation. We modified the figure, overlapping the points with a density plot that emphasizes more clearly the actual distribution of points. We believe in the new plot the correlation is more clear (Figure below left, and new Figure 3C). To answer the question regarding the role of the specific three points indicated by the reviewer, we found that they decrease, rather than contribute to, the negative correlation. When we remove them (by removing all points above 60 minutes), the correlation goes from -0.23 to -0.28.

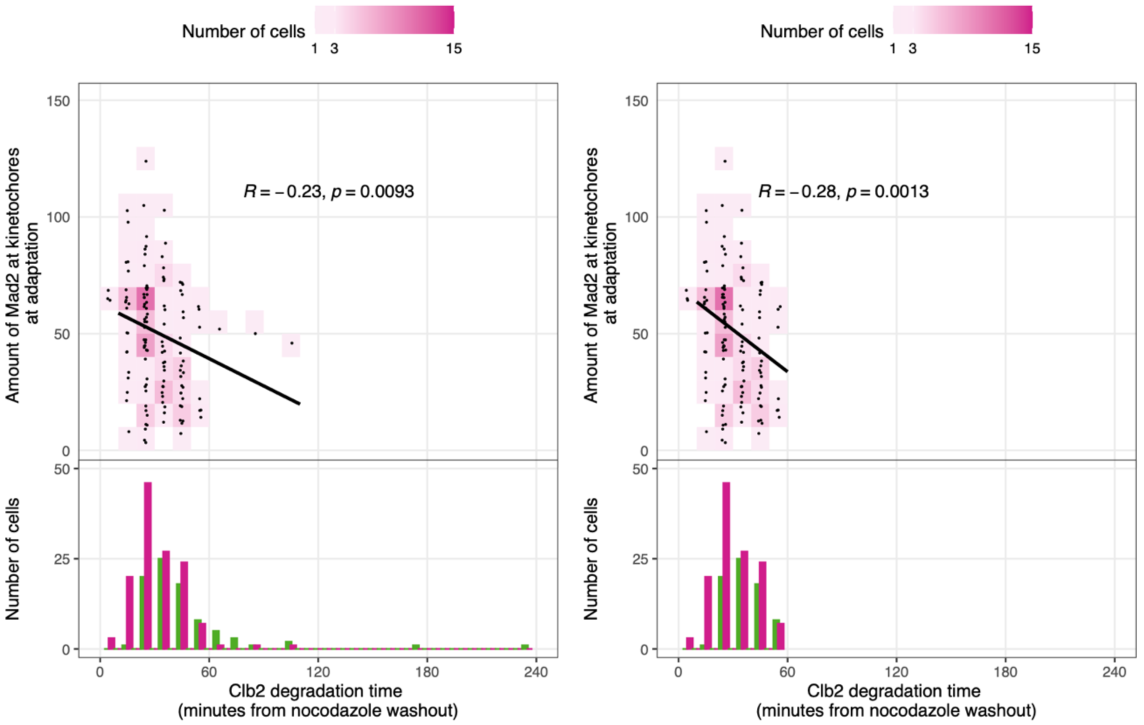

Given this result, we kept Mad2 intensity as a proxy for unattached kinetochores.

4. The proposed model suggests that fluctuations in APC/C-Cdc20 activity destabilise the checkpoint. Could these fluctuations be buffered by increasing ploidy, thereby reducing noise? Exploring or at least discussing this possibility might add depth to the paper. If this were the case, would the same noise exist in mammalian systems? A brief discussion on the role of noise and stochastic processes in cell fate decisions between the two systems would strengthen the manuscript.

The point raised by the reviewer is interesting. The intuition that noise in gene expression is reduced in diploid cells applies to intrinsic noise due to variations in molecular number (see Di Talia et al, Nature, 2007, PMID: 17713537). However, if we assume that extrinsic variations in transcription are the dominant form of noise, as we do in our model, then things might be different. As Becskei et al. have shown, variations in the expression of different

copies of the same gene can be highly correlated in yeast, resulting in a negligible reduction in noise levels (Becskei, Kaufmann and van Oudenaarden, Nat Gen, 2005, PMID: 16086016. Hence, it is difficult to produce a clear prediction for yeast diploids.

The question, however, is not so much about yeast diploids but about mammalian cells. How much of what we describe in the paper applies to mammalian cells is a very relevant point, which we covered in the Discussion. We have expanded it, to discuss both similarities and differences between the two systems. One major similarity regards the molecular players and the role of stochasticity. One relevant difference is the dynamics of Cyclin B during the arrest. In yeast, the protein is stable, and suddenly degraded when cells adapt. In mammals, it is slowly degraded until cells adapt, when again it is quickly degraded. It is still possible to reconcile our framework with mammalian cells as follows: fluctuations in APC/C^{Cdc20} contribute to the continuous degradation of the cyclin. This could in turn accelerate the rate of adaptation. If this were the case, also in mammalian cells adaptation would be a major consequence of the steady state trap. This argument is now given in the expanded Discussion.

5. In the introduction, the manuscript states, "After adaptation, chances are that cells missegregate chromosomes." If this observation has been documented previously, a reference should be included. If it is speculation or a finding of the current study, consider moving this statement to the discussion section for clarity.

We reformulated the statement, making it more hypothetical: "Since cells progress into anaphase when microtubules and kinetochores are properly attached, adaptation is at risk of eliciting chromosome missegregation and aneuploid progeny." This, we believe, is a conjecture which belongs to the Introduction, since it puts forward the potential risks of adaptation. It also relates with the analysis of the causal relation between adaptation and missegregation which, following a suggestion of the reviewer, we introduced among the results (new panel Figure S5H).

6. Please elaborate on possible mechanisms by which Mps1 overexpression activates the checkpoint in anaphase. This would help contextualise the role of Mps1 in the positive feedback loop.

In early anaphase, kinetochores are still attached to microtubules, but there is no tension between sister chromatids since cohesin has been cleaved. In this context, Mps1 may reactivate the checkpoint since it plays a role in the checkpoint but also contributes to error correction, a process which is triggered by the lack of tension (see for example Benzi et al, EMBO Rep, 2020, PMID: 32307893). We added this notion in the revised manuscript.

7. In the introduction, define "PFL" (I assume it stands for Positive Feedback Loop) for clarity, as it may not be immediately familiar to all readers.

Done.

8. The phrase "cells are trapped in a condition that favours entry in anaphase with unattached kinetochores, even with the drug is washed out" should be revised. Instead of

*"even", consider using "especially" or *"even more" to accurately reflect that washing out nocodazole increases the frequency of missegregation.*

Done.

9. Provide more detail about the APC-A mutant. Specifically, which subunits are mutated and at which residues?

Two subunits are mutated: Cdc16 (Apc6 in mammals) and Cdc27 (Apc3 in mammals). More precisely, all serine/proline (SP) and threonine/proline sites (TP) on Cdc16 and Cdc27 were mutated to alanine. For CDC16, SP are in position 44, 59, 95 and 103, while TP are in position 115 and 406. For CDC27, SP are in positions 267 and 328, while TP are in positions 304, 351 and 397. These mutants were produced by Rudner and Murray (JCB, 149(7), 1377-1390, 2000). We added these details when we present the mutant APC-A.

In conclusion, this manuscript presents a novel perspective on mitotic checkpoint adaptation, particularly highlighting the increased risk of chromosome missegregation during nocodazole washout. While the work is scientifically robust and overall of excellent quality, greater emphasis on the key findings and better organisation of the results would significantly enhance its impact. Despite some presentation issues, this study provides interesting new insights into the dynamics of checkpoint adaptation and its link to aneuploidy.

Packed model

```
kr=kb*KD
kcat=kact*k_nuk
k1=(kcat+km1+kdeph)/J
kpho=kdeph/kph

#conservations
a=apctot-(ac+acmc)
m=mad2tot-(mstar+mc+acmc)
ct=c+mc+acmc*2+ac

Mps1_new=Mps1/(Mps1+kph)

#odes
dMps1/dt=ksyn_mp-kdeg_mp*Mps1*ac-kdegbg_mp*Mps1
dc/dt=ksyn_c20-kassmc*mstar*c+kdissmc*mc-kassac*a*c+kdissac*ac+kdeg*acmc-
kdegbg*c
dmstar/dt=(kcat*m*St)/(J+m)*(Ut*Mps1_new)/(KD*(J/(m+J+kdeph/kb))+Ut)-kinact*mstar
-kassmc*mstar*c+kdissmc*mc
dmc/dt=kassmc*mstar*c-kdissmc*mc-kassacmc*ac*mc+kdissacmc*acmc-kdegbg*mc
dac/dt=kassac*a*c-kdissac*ac-kassacmc*ac*mc+kdissacmc*acmc-kdegbg*ac
dacmc/dt=kassacmc*ac*mc-kdissacmc*acmc-kdeg*acmc-kdegbg*acmc

#parameters
p St=25
p Ut=32
p ksyn_c20 = 42.2658
p ksyn_mp = 11.2849
p kdegbg = 0.03808
p kinact = 0.00004751
p J = 2.1127
p kact = 0.0242
p k_nuk = 735
p KD = 4.5
p kassmc = 0.0215
p kdissmc = 0.0313
p kassac = 0.1073
p kassacmc = 0.5794
p kdissac = 0.9414
p kdissacmc = 0.6115
p kdeg = 1.3127
p kdeg_mp = 0.0240
p kdegbg_mp = 0.0617
p k=10
p kb=1000
p km1=10
p kph=100
p kdeph=10

# totals
```

p apctot = 100
p mad2tot = 175

#initial conditions
i mps1=17
i c=13
i mstar=136
i mc=4
i ac=26
i acmc=29

@ total=2000, boundaries=10000
#AUTO settings
@ dsmax=.5, dsmin=.0001, ds=-.02, ntst=1500, nmax=2000, npr=500, parmin=0, parmax=32
@ autoxmin=0, autoxmax=32, autoymin=0, autoymax=100
done

unpacked model

kr=kb*KD
kcat=kact*k_nuk
k1=(kcat+km1)/J
kpho=kdeph/Kph

#conservation relation and definition of new variables
a=apctot-(ac+acmc)
m=mad2tot-(mstar+USM+mc+acmc)
ct=c+mc+acmc*2+ac
U=Ut-USM-US
Si=St-(USM+US+S)

#odes
dMps1/dt=ksyn_mp-kdeg_mp*Mps1*ac-kdegbg_mp*Mps1
dc/dt=ksyn_c20-kassmc*mstar*c+kdissmc*mc-kassac*a*c+kdissac*ac+kdeg*acmc-kdegbg*c
dmstar/dt=kcat*USM-kinact*mstar-kassmc*mstar*c+kdissmc*mc
dmc/dt=kassmc*mstar*c-kdissmc*mc-kassacmc*ac*mc+kdissacmc*acmc-kdegbg*mc
dac/dt=kassac*a*c-kdissac*ac-kassacmc*ac*mc+kdissacmc*acmc-kdegbg*ac
dacmc/dt=kassacmc*ac*mc-kdissacmc*acmc-kdeg*acmc-kdegbg*acmc
dUS/dt=kb*U*S-kr*US-k1*US*m+km1*USM+kcat*USM-kdeph*US
dUSM/dt=k1*US*m-km1*USM-kcat*USM-kdeph*USM
dS/dt=kpho*Si*Mps1-kdeph*S-kb*U*S+kr*US

#parameters
p St=25
p Ut=32
p ksyn_c20 = 42.2658
p ksyn_mp = 11.2849
p kdegbg = 0.03808
p kinact = 0.00004751

```
p J = 2.1127
p kact = 0.0242
p k_nuk = 735
p KD = 0.1
p kassmc = 0.0215
p kdissmc = 0.0313
p kassac = 0.1073
p kassacmc = 0.5794
p kdissac = 0.9414
p kdissacmc = 0.6115
p kdeg = 1.3127
p kdeg_mp = 0.0240
p kdegbg_mp = 0.0617
p k=10
p kb=1000
p km1=10
p Kph=100
p kdeph=10

# totals
p apctot = 100
p mad2tot = 175


#initial conditions
i Mps1=17
i c=13
i mstar=136
i mc=4
i ac=26
i acmc=29
i us=1
i usm=2
i s=2


@YP=AC,TOTAL=200,METH=cvode,atol=3e-10,toler=3e-
10,XHI=200,YLO=0,BOUND=10000,maxstorage=100000
#AUTO settings
@ dsmax=.5, dsmin=.0001, ds=-.02, ntst=1500, nmax=2000, npr=500, parmin=0, parmax=32
@ autoxmin=0, autoxmax=32, autoymin=0, autoymax=100
done
```
