# Supplementary material for: lncreased risk of slippage upon disengagement of the mitotic checkpoint
Source: PLoS Comput Biol. 2025 Mar 19;21(3):e1012879. doi: 10.1371/journal.pcbi.1012879 (PMC11981154; doi:10.1371/journal.pcbi.1012879)
Supplement: Attachment — Submitted filename: Review PCOMPBIOL.docx [file pcbi.1012879.s011.docx]

Review PCOMPBIOL-D-24-01748

In this paper, Stier et al. study the phenomenon of adaptation to (‘slippage from’) the spindle assembly checkpoint (SAC) in budding yeast cells, by a combination of judicious experiments and careful computational modeling. In my opinion, the experiments and modeling are done very professionally, and they provide new insights into the mechanisms whereby yeast cells bypass the SAC and exit mitosis with chromosomes that are not properly aligned on the mitotic spindle. This is an important problem in cell biology, with implications for carcinogenesis in human cells. I recommend the paper for publication in PLOS Comp Biol after minor revisions.

I have a few comments and suggestions that should be addressed in a revision:

1. In the Abstract, the authors say, “According to the model, drug removal further facilitates adaptation, a prediction we confirmed in budding yeast.” That is true: the computational model predicts that removing the checkpoint signal (nocodazole) causes more cells to enter anaphase with unaligned chromosomes (adaptation) than would happen with the checkpoint in place, and the authors confirmed this prediction. But, on p 9, when introducing these simulations and experiments, the authors say, “We reasoned that dismantling the checkpoint by washing out nocodazole may achieve this goal” (i.e., the goal of “entering anaphase in the presence of unattached kinetochores”). They don’t explain their reasoning. Why is it counterintuitive to think that, once the checkpoint is engaged by nocodazole and many chromosomes are unattached to the spindle, removing the checkpoint inducer would increase the probability of slippage? What causes them to think removing the signal would decrease slippage? In the following paragraph, they say, “In contrast to expectation (i.e., to their reasoning), the majority of cells (60.8%) started Clb2 degradation in the presence of localized Mad2 even after washout.” Should we be surprised? Their model simulations (Fig. S3C) don’t help: during normal exit, fluctuations don’t flip the checkpoint OFF until nearly all the kinetochores are aligned, but during adaptation, fluctuations flip the checkpoint OFF with many (or a few) unaligned kinetochores. What’s the difference in these simulations? What do they have to do with removing nocodazole? Since these experiments and simulations are a major conclusion of the paper, they must be explained more clearly.

2. I also have some issues with the discussion of the “steady-state trap”. It is true that, during normal cell cycles (in absence of nocodazole), the frequency of mis-segregation is very low, but in response to nocodazole treatment (sustained or temporary), the frequency of mis-segregation is high. Furthermore, during normal cycling, the SAC genes are dispensable, presumably because there is plenty of time for chromosomes to be properly aligned on the spindle before other restraints on APC/C activation are lifted. As the authors say, “the mitotic checkpoint is only transiently, if at all, activated during a regular yeast cell cycle.” The checkpoint mechanism, once engaged, “stalls cell division, but it struggles to keep APC/CCdc20 levels under control.” That is true, since spontaneous fluctuations may kick a cell out of the domain of attraction of the checkpoint ON state. Whether or not adaptation occurs depends on the height of the barrier (about 20 molec/cell in Fig. S3C) and the number of molec of APC/CCdc20 in the ON state (~25 in Fig. S3C). If fluctuations are Poisson, then their std dev is 5 molec and an excursion of 20 molec should be quite rare; correct? Are these the fluctuations we are seeing in Fig. S3C?

I suspect that “adaptation” has a selective advantage in yeast cells. If, for any reason, a yeast cell gets stalled in mitosis by the SAC, its best strategy may be to slip out of the checkpoint and “take its chances” to produce viable progeny rather than to remain stuck in mitosis. Of course, in multicellular organism, such slippage can be catastrophic (carcinogenic).

3. Table 1: the authors mean molec/nucleus rather than molec/cell.

4. Table 2: why are some parameter values specified to 6 significant figures (16.9063 nM/min) and others to only one (0.03 /min)?

5. Table 3: Madi → Mada rate is described by Michaelis-Menten kinetics, but MM kinetics is notoriously suspect in stochastic calculations, as the senior author knows well. A word of explanation may be in order.

6. The authors are inconsistent in notation: sometimes APC/CCdc20 and sometimes APC/C^Cdc20^. I prefer the latter, or APC/C-Cdc20. But be consistent.

7. The authors should make all computer code publicly available.
